# Design and Application of Strong and Tough Low-Friction Hydrogels

**DOI:** 10.3390/gels12090775

**Published:** 2026-08-30

**Authors:** Xian Wei, Hongli Luo, Jiangze Luo, Dongya Zhang, Bo Huang, Youjing Liu, Ziling Xu, Ruchao Kou

**Affiliations:** 1 School of Smart Manufacturing, Panzhihua University, Panzhihua 617000, China; 2 School of Mechanical Engineering, Xi’an University of Technology, Xi’an 710048, China

**Keywords:** strong and tough low-friction hydrogels, hydration lubrication, functional decoupling design, double-network hydrogels

## Abstract

Hydrogels, with their high water content, tissue-like softness, and excellent biocompatibility, are prime candidates for dynamic load-bearing interfaces such as cartilage replacement and implant coatings. However, the toughening structures introduced to enhance damage resistance often compromise surface lubrication: highly dissipative networks, while suppressing bulk crack propagation, frequently increase interfacial friction and accelerate wear. This toughness–lubrication trade-off constitutes a central bottleneck limiting the long-term service of hydrogels under dynamic contact conditions. This review examines the friction and wear behavior of various hydrogel systems and, from the dual perspectives of bulk mechanical reinforcement and surface lubrication regulation, summarizes the core design mechanisms of toughening and hydration lubrication strategies, respectively. Based on this analysis, this review proposes a functional decoupling design principle: hierarchical structures—ranging from homogeneous to heterogeneous—in which the bulk dissipates mechanical load while the surface maintains hydration lubrication, thereby reconciling mechanical toughness with lubrication. Finally, this review surveys cutting-edge applications of such materials in tissue engineering, device coatings, drug delivery, electronic energy-harvesting and storage devices, and soft actuators, providing a reference for the development of hydrogels that integrate excellent mechanical properties with lubrication functionality.

## 1. Introduction

Hydrogels are soft materials consisting of hydrophilic polymer chains that form three-dimensional networks, capable of absorbing and retaining substantial amounts of water without dissolving [1]. They uniquely combine the structural stability of solids with the permeation and transport properties of liquids. In 1960, Wichterle and Lim [2] published a seminal paper reporting the first synthesis of crosslinked poly(2-hydroxyethyl methacrylate) (pHEMA) hydrogels, whose tunable mechanical properties and excellent biocompatibility garnered widespread attention and formally established modern hydrogel research. The evolution of hydrogels has progressed through three principal stages. First-generation hydrogels relied primarily on covalent chemical crosslinking, where crosslinking agents form irreversible linkages to construct permanent three-dimensional networks. These materials exhibited high water absorption and basic elasticity [3,4,5], yet suffered from low mechanical strength and irreversible structures. Building upon chemical crosslinking and inspired by Tanaka’s phase transition theory [6], second-generation hydrogels introduced stimuli-responsive characteristics, enabling them to sense external changes in temperature, pH, or ionic strength and undergo reversible alterations in volume or properties. Representative examples include poly(N-isopropylacrylamide) (PNIPAM) thermosensitive gels [7] and calcium alginate ion-responsive gels [8], which marked a leap from passive materials to “intelligent” controlled-release platforms. These hydrogels have found broad applications in sensing [9], biomimetic muscles [10], tissue engineering [11], and drug delivery [12]. Third-generation hydrogels integrate supramolecular interactions, dynamic covalent bonds, and nanocomposite technologies, imparting combined attributes such as high strength, self-healing [13], injectability [14], bioactivity [15], and multi-responsiveness [16]. They demonstrate diversified value in biomedicine, encompassing controlled drug delivery systems [17], tissue regeneration scaffolds [18], in vitro physiological model construction [19], in vitro cell culture platforms [20], in vivo implant materials [21], wound repair dressings [22], surface modification coatings for medical devices [23], and corneal contact lenses for vision correction [24]. Moreover, hydrogels have attracted substantial interest in emerging fields such as flexible electronics and intelligent soft robotics, with specific frontier applications spanning flexible sensing elements [25], artificial muscle actuators [26], biomimetic soft robotic systems [27], stretchable electronic devices [28], flexible energy storage batteries [29], high-power supercapacitors [30], water purification membranes [31], ionic electronic devices [32], magnetically responsive smart materials [33], optical devices [34], acoustic functional materials [35], underwater tough adhesives [36], and functional protective coatings [37].

For hydrogels deployed in biomedical sliding interfaces—such as joint replacement and tissue engineering—concurrent mechanical robustness and low friction are essential: they must integrate strength and toughness comparable to those of native tissues to withstand complex physiological loads, while simultaneously exhibiting extremely low friction coefficients to ensure long-term, stable, and painless motion. However, these two attributes inherently trade off in traditional material design. High strength and toughness typically rely on dense crosslinking networks or efficient energy dissipation mechanisms [38,39], yet such strategies inevitably increase internal friction, hysteresis, and surface roughness. In stark contrast, excellent lubrication requires a highly hydrated [40], soft, and smooth surface, which often compromises load-bearing capacity. Consequently, reconciling toughness with lubricity remains a central challenge, and considerable effort has been directed toward designing tough, low-friction hydrogels. To address this dilemma, researchers have developed a variety of engineering strategies. For instance, double-network (DN) structures [41]—where fracture of a stiff, brittle first network dissipates energy while a soft, ductile second network accommodates large deformation—have successfully elevated hydrogel toughness to values on par with rubber and even cartilage. However, this toughening mechanism is typically accompanied by pronounced internal friction and hysteresis; without deliberate surface engineering, fluid-film lubrication remains elusive. Another effective strategy incorporates zwitterionic polymers [42], whose exceptionally strong electrostatically induced hydration forms a dense hydration layer at the surface, thereby achieving ultra-low friction coefficients under boundary lubrication conditions. Yet, pure zwitterionic gels generally suffer from weak mechanical properties and poor tear resistance. Furthermore, although nanocomposite or topological structures can partially balance mechanical and lubrication properties [43,44], how to systematically integrate these attributes so that the materials simultaneously deliver high load-bearing capacity and low friction under complex mechanical environments remains an open question.

To avoid terminological overgeneralization and ensure that the review scope remains operationally well-defined, this review strictly defines “tough, low-friction hydrogels” as hydrogel systems that simultaneously satisfy the following performance criteria: (1) bulk mechanical robustness comparable to or superior to that of natural load-bearing soft tissues—specifically, tensile or compressive strength on the order of megapascals and fracture energy on the order of 10^3^ J m^−2^, thereby ensuring structural integrity at load-bearing interfaces; and (2) interfacial lubricity under aqueous boundary or mixed lubrication—specifically, a steady-state coefficient of friction below ~0.1. On this basis, representative studies are not selected by material composition or structure alone; instead, two inclusion principles are applied. First, the system must demonstrate a discernible synergistic or decoupled pathway between mechanical reinforcement mechanisms and lubrication functionality. Second, all performance data must derive from reproducible, standardized tests (e.g., ball-on-disk reciprocating friction, uniaxial tension) to ensure valid cross-system comparisons.

Despite substantial progress in designing tough, low-friction hydrogels, the intrinsic interplay among the various toughening and lubrication mechanisms remains poorly understood. Notably, hydrogels with similar chemical compositions and microstructures can exhibit markedly different load-bearing capacities and tribological properties, revealing a fundamental question: similar compositions and structures do not guarantee consistent synergistic mechanisms of toughness and lubrication. Current optimization strategies—such as modulating energy dissipation through sacrificial bond networks or enhancing hydration lubrication via surface-bound lubricating moieties—largely target individual mechanisms in isolation, without accounting for multi-mechanism coupling effects. Consequently, there is a pressing need to establish universal design principles grounded in a hierarchical logical framework that can guide diverse hydrogel systems toward synergistic enhancement of toughness and lubrication.

**Figure 1:**
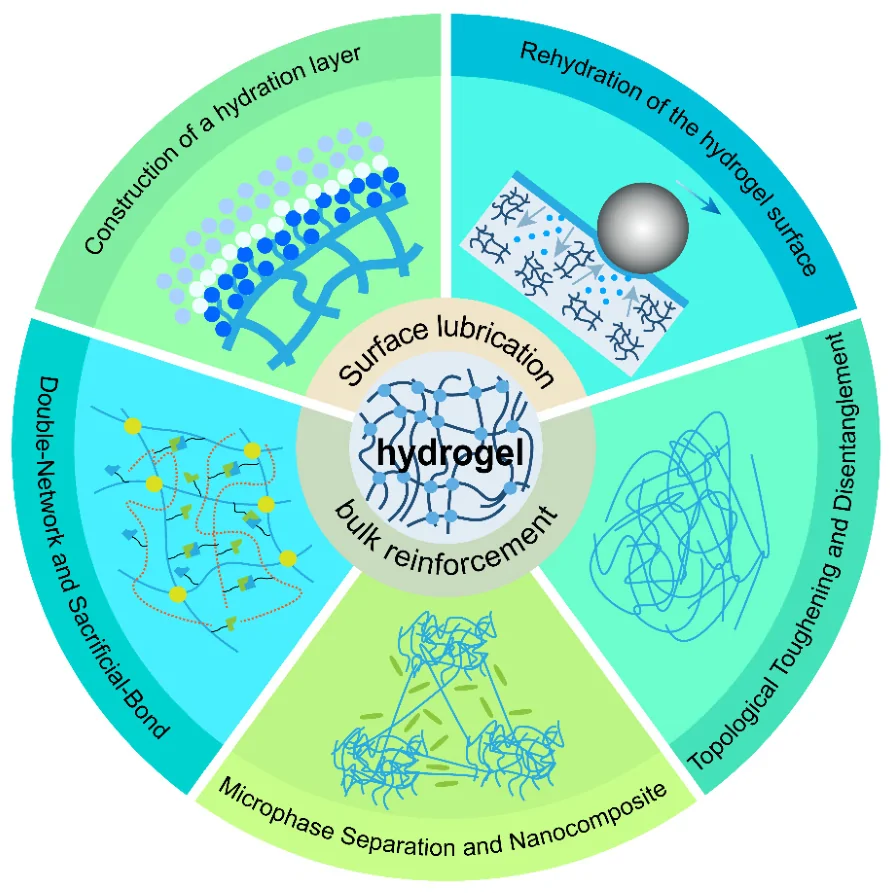
Design Strategies for Tough, Low-Friction Hydrogels. Line, polymer network chain; blue spheres, water molecules; yellow spheres, chemical crosslinking points; large gray/white spheres, counterface (friction pair); irregular polygons, sacrificial bonds (reversible or irreversible energy-dissipation units); green rods, nanofillers; arrows, direction of motion.

A thorough understanding of current strategies for designing tough, low-friction hydrogels is essential for developing advanced materials with superior performance. In this review, we first outline recent advances in tough, low-friction hydrogels and then provide a systematic classification of the underlying mechanisms based on composition and structure (Figure 1). Building on a comprehensive analysis of existing toughening and lubrication strategies, we propose overarching principles for synergistically integrating toughness with low friction. We further summarize and critically discuss the key functional properties of such hydrogels reported in the literature and their prospects for cross-disciplinary applications. Finally, we identify current research limitations and offer a forward-looking perspective on future directions and potential breakthroughs.

## 2. Tribological Behavior and Failure Characteristics of Toughened Hydrogels

Before examining specific toughening and lubrication strategies, it is essential to understand how these hydrogels perform under sliding contact. Their friction, wear, and failure modes during prolonged sliding provide the critical experimental basis for rational design.

### 2.1. Basic Methods for Tribological Testing of Hydrogels

The tribological characterization of hydrogels presents unique challenges owing to their high water content and soft nature, including large contact deformation, nonlinear load–contact area relationships, and a tendency for the surface hydration state to drift. Common test configurations that have been developed to address these challenges include: ball/pin-on-disk tests, where a stainless steel, SiC, or glass hemisphere counterface slides against the hydrogel under submerged or wetted conditions to measure friction force as a function of load and sliding speed; in situ observation tests, which monitor the contact state and lubrication regime transition at a transparent glass substrate–hydrogel interface in real time [45]; and biotribological tests that replicate the oscillatory or reciprocating contact motions of natural joints.

Early investigations revealed that hydrogel friction deviates markedly from the classic Amontons’ law: the friction coefficient typically decreases with increasing load, responds nonlinearly to sliding speed, and depends critically on the surface hydration state [46]. This unconventional frictional behavior indicates that the lubrication mechanisms in hydrogels are fundamentally distinct from those in hard materials.

### 2.2. Exploration of Friction and Wear Behavior in Toughened Hydrogels

#### 2.2.1. Single-Network and Double-Network Hydrogels

**Figure 2:**
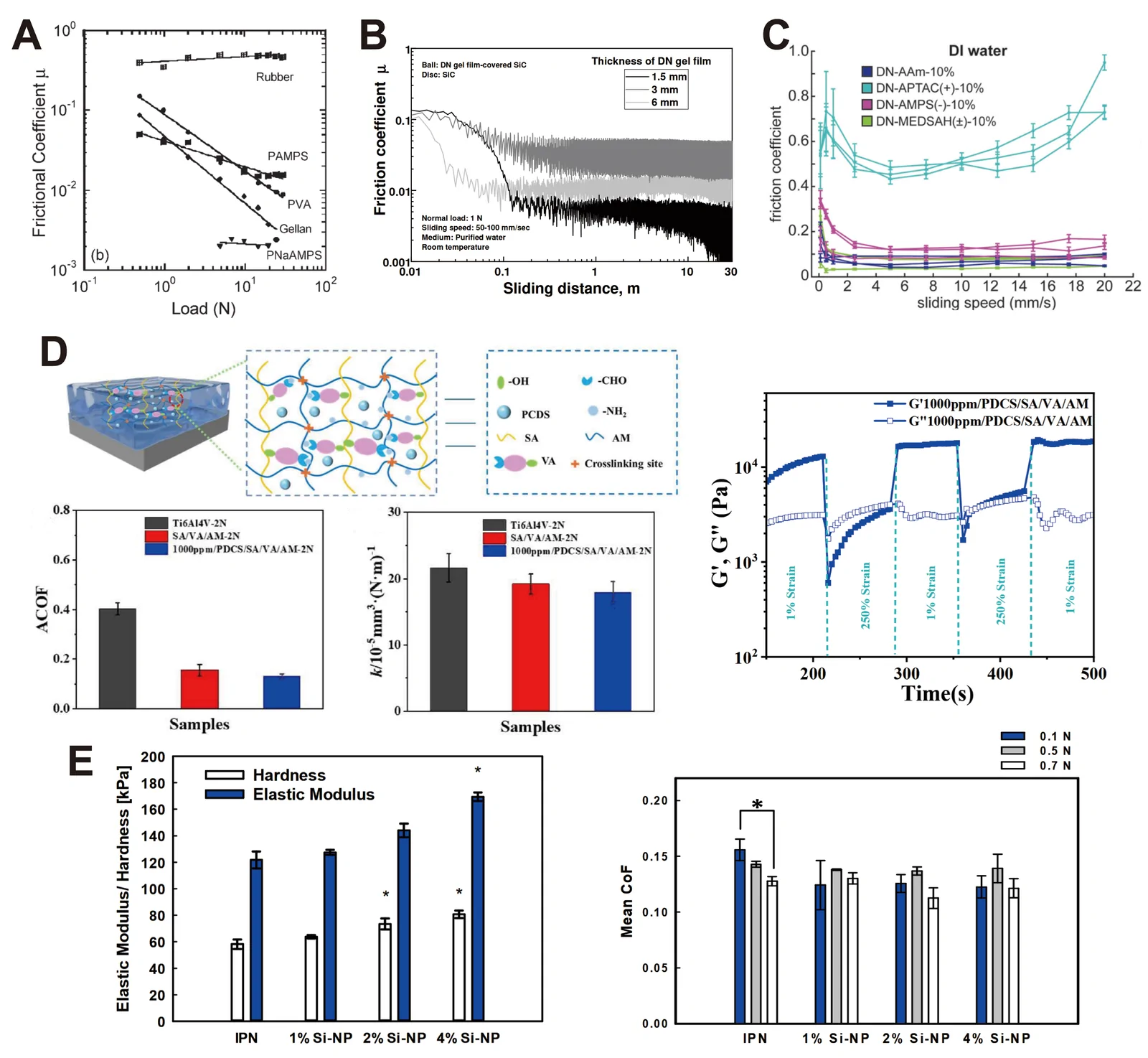
Friction and wear behavior of toughened hydrogels. (**A**) Single-network PAAm: COF ~0.001 under moderate loads. Reproduced with permission [46]. Copyright 2006, Royal Society of Chemistry. (**B**) DN films: 1.5 mm reaches μ < 0.01 after two running-in cycles; 6 mm shows gradual decrease. Reproduced with permission [49]. Copyright 2021, Elsevier. (**C**) DN with neutral/zwitterionic second networks yield lowest COF. Reproduced with permission [50]. Copyright 2021, Elsevier. (**D**) Self-healing Schiff-base DN: COF 0.13 at 2 N, low wear rate. Reproduced with permission [52]. Copyright 2025, MDPI. (**E**) Silica nanocomposite: COF 0.0035-0.0055, 69% wear reduction (serum); high loading increases wear under dry sliding. * indicate statistically significant differences compared to the control IPN hydrogel (*p* < 0.05). Reproduced with permission [53]. Copyright 2019, Elsevier.

Single-network hydrogels, consisting of a single polymer network crosslinked into a three-dimensional architecture, represent the most fundamental hydrogel system. Gong et al. [46] systematically investigated the sliding friction of single-network hydrogels and demonstrated that, under moderate loads (e.g., normal load of 0.1–10 N, sliding velocity of 7 mm min^−1^ against a smooth glass substrate in air), the gel surface can sustain an ultra-low coefficient of friction (COF as low as ~0.001)—far below typical values for conventional solid friction pairs (Figure 2A). In polyacrylamide (PAAm) single-network hydrogels, the high segmental mobility of hydrated polymer chains, together with the hydration repulsion layer formed at the surface, effectively minimizes interfacial shear resistance. However, this low friction is intrinsically coupled with low modulus and poor load-bearing capacity. As the normal load increases, the gel network undergoes large deformation, the contact area increases sharply, and the wear rate accelerates markedly. Tribological studies on poly(vinyl alcohol) (PVA) hydrogels further reveal that single-network gels experience continuous wear and material detachment during sliding; the resulting wear debris may temporarily function as a “third-body lubricant,” but over prolonged cycling, irreversible mass loss predominates [47]. Consequently, while single-network hydrogels can exhibit ultra-low friction under limited loads, their inherent mechanical fragility severely restricts long-term tribological performance.

The advent of double-network (DN) hydrogels substantially extended the mechanical performance limits of hydrogels. Yasuda et al. [48] used a pin-on-flat reciprocating apparatus with an alumina ceramic pin (diameter 9 mm, spherical end, surface roughness Ra ≈ 0.006 μm) in distilled water at 20 °C, under a normal load of 4.7 N (corresponding to a nominal contact pressure of 0.1 MPa), a stroke length of 25 mm, a frequency of 1.0 Hz (average sliding speed 50 mm/s), and for 10^6^ cycles (total sliding distance 50 km) to evaluate the wear resistance of four DN hydrogels and found that the poly(2-acrylamido-2-methylpropanesulfonic acid)-poly(N,N-dimethylacrylamide) (PAMPS-PDMAAm) DN gel exhibited wear resistance comparable to that of ultra-high molecular weight polyethylene (UHMWPE). Importantly, the study revealed that wear resistance quantifies macroscopic volume loss, whereas the friction coefficient reflects interfacial shear behavior, and the two do not necessarily correlate. Jay et al. [49] investigated the effect of film thickness on the tribological behavior of DN hydrogels (Figure 2B) under water-lubricated sliding against a SiC disc: a 1.5 mm thick DN film entered an ultralow friction regime (μ < 0.01) after just two run-in cycles, with the coefficient of friction (COF) subsequently stabilizing, whereas a 6 mm thick film displayed a friction curve that decreased only gradually. This thickness-dependent behavior indicates that the friction of DN hydrogels is strongly governed by the contact-area evolution driven by elastic deformation. Bonyadi et al. [50] characterized the tribology of DN hydrogels bearing second networks with distinct charge characteristics (Figure 2C) under reciprocal sliding against a 2 mm diameter steel ball probe in both deionized water and fetal bovine serum (FBS), with speeds spanning 0.1 to 20 mm/s and a constant normal load of 10 mN, and found that neutral or zwitterionic second networks yielded the lowest COF and shear stress—values markedly lower than those of natural articular cartilage. This finding highlights that the chemical nature of the second network, particularly its hydration capacity and surface charge state, critically governs the interfacial hydration lubrication efficiency, largely independently of the bulk mechanical properties. Consequently, the intrinsic decoupling between surface lubrication and bulk mechanics in DN gels offers a powerful lever for reconciling toughness with low friction.

The toughening conferred by double-network (DN) structures, however, comes at the expense of a friction penalty. Under sliding shear, the stiff and brittle first network fractures irreversibly; the resulting debris accumulates at the interface, driving a progressive increase in the coefficient of friction over extended cycling. This debris-mediated mechanism is inferred from the established bulk fracture behavior of DN gels, in which the brittle first network sacrificially fragments into microscopic clusters ahead of the crack tip [48,51]. While Yasuda et al. [48] confirmed the excellent wear resistance of DN gels under 10^6^ cycles and Jay et al. [49] elucidated thickness-dependent elastic effects on run-in friction, neither study directly isolated the contribution of interfacial debris accumulation to friction escalation. The susceptibility of the first network to fragmentation under shear nevertheless provides a physically grounded rationale for the friction penalty and its expected worsening with prolonged cycling. Moreover, the dense network of sacrificial bonds amplifies internal friction and hysteresis, rendering DN hydrogels—unless deliberately surface-engineered—largely incapable of sustaining a stable fluid-film lubrication regime under high loads.

#### 2.2.2. Dynamic-Bond and Self-Healing Hydrogels

Self-healing hydrogels, capable of autonomously restoring structural integrity after damage through dynamic bonds, offer a compelling strategy to combat wear in sliding interfaces. This self-healing functionality originates from dynamic covalent or supramolecular bonds that undergo reversible dissociation and re-association in response to stimuli (e.g., pH, temperature, light), thereby repairing damage akin to biological tissues and substantially prolonging material service life. Li et al. [52] developed a self-healing double-network hydrogel coating based on Schiff base dynamic covalent bonds and hydrogen bonds (Figure 2D). During reciprocating sliding against a Si_3_N_4_ ball in simulated body fluid (SBF) at room temperature, with a frequency of 1 Hz, a stroke length of 4 mm, and a test duration of 1800 s, the reversible dissociation–reassociation of the Schiff base linkages imparted a dynamic repair capability to the coating, enabling real-time healing of the worn surface. Under a load of 2 N, the average coefficient of friction (COF) was 0.13, and the wear rate remained consistently low. The rapid reorganization of dynamic bonds served as an interfacial self-healing mechanism, partially offsetting wear-induced material loss. Yang et al. [54] introduced dynamic B–O coordination bonds into a sodium alginate matrix and, through glycerol–ethanol solvent exchange, modulated the bound-to-dissociated ratio of these bonds, thereby achieving reversible tuning of the hydrogel’s mechanical modulus and dynamic switching of the COF between 0.022 and 0.157. This work reveals that the tribological influence of dynamic bonds is governed not only by bond strength and reversibility, but also critically by the swelling state and solvent environment. Consequently, self-healing hydrogels provide a unique route where interfacial damage can be continuously repaired during sliding, yet their tribological performance remains intimately coupled to the environmental responsiveness of the dynamic chemistries employed.

Although self-healing hydrogels can partially offset wear accumulation through dynamic bond reorganization, translating bulk self-healing to a sliding interface introduces distinct kinetic challenges: bond cleavage is driven by high local shear stress concentrated at the contact zone, while re-formation requires adequate time and favorable environmental conditions (pH, solvent composition, temperature) that may not be maintained under continuous sliding. Evidence from Schiff-base [52] and boronate-ester [54] systems under reciprocating wear tests indicates that interfacial repair can fall behind material removal under the examined loads and speeds. While it remains to be determined whether faster dynamic chemistries (e.g., autonomously catalyzed or photo-triggered systems) can close this gap, current results suggest that relying on network-level dynamic bonds alone is unlikely to fully resolve friction escalation during prolonged sliding.

#### 2.2.3. Nanocomposite and Fiber-Reinforced Hydrogels

Nanocomposite reinforcement and fiber reinforcement represent two core strategies for pushing the mechanical limits of hydrogels beyond those of homogeneous networks. Nanocomposite hydrogels incorporate nanoparticle fillers that form dynamic sacrificial bonds (e.g., hydrogen and ionic bonds) with the surrounding polymer chains; under load, these bonds reversibly rupture and re-form, dissipating energy and deflecting cracks to toughen the network [55]. Fiber-reinforced hydrogels, in contrast, embed micro- or nanoscale fibrous skeletons into the matrix [56]. Toughening in these systems relies on load transfer, crack bridging, and fiber pull-out and fracture, which substantially boost strength and modulus, while controlled fiber alignment further enables anisotropic, biomimetic architectures.

Arjmandi et al. [53] investigated the mechanical and tribological behavior of a silica nanoparticle nanocomposite hydrogel (Figure 2E) under reciprocating sliding against a 3 mm diameter alumina ceramic ball at normal loads of 0.1–0.7 N, sliding speeds of 50–150 mm/s, and a total sliding distance of 1000 m. Under bovine serum lubrication, an optimal nanofiller loading reduced the wear volume by over 69% and lowered the coefficient of friction (COF) to 0.0035–0.0055. Strikingly, under dry sliding, samples with a high nanoparticle concentration (4 wt%) exhibited greater wear than other samples, and the addition of nanoparticles produced no statistically significant reduction in COF—revealing that the tribological benefits of nanofillers are strongly lubrication-dependent.

To circumvent the trade-off that a homogeneous network must simultaneously manage interfacial lubrication and bulk load-bearing within the same material region, Sun et al. [57] adopted a cartilage-inspired hierarchical design that integrates a lubricious surface layer with a tough, fiber-reinforced core. The loosely packed open network of the surface layer accommodates water for hydration lubrication, while the folded peptide fibers in the core bear the majority of the normal load, suppressing surface damage caused by plastic deformation of the contact area during sliding. When subjected to reciprocating sliding against a Ti6Al4V ball under a 5 N load at 0.5 Hz in simulated body fluid, this coating sustained a COF of ≈0.009 and high wear resistance over 100,000 sliding cycles. By partitioning lubrication and load-bearing into distinct structural layers, the design decouples the conflicting requirements of interfacial hydration and bulk toughness.

In a similar hierarchical vein, Liu et al. [58] fabricated a concrete-like hydrogel using PVA particles as aggregate and PVA/polyethylene glycol (PEG) fibers as reinforcing fibers. The composite achieved a compressive strength of 29.5 MPa and a tensile strength of 10.5 MPa, while maintaining low friction and minimal wear under submerged ball-on-disk testing against a stainless-steel counterface (load: 1–10 N; frequency: 1–5 Hz; lubricants: physiological saline, PBS, and a hyaluronic acid/plasma mixture). This demonstrates that macroscopic fiber-reinforced designs can concurrently deliver high load-bearing capacity and underwater lubricity.

Collectively, the experimental evidence exposes a fundamental tension between toughening and friction reduction. Double-network strategies that rely on irreversible covalent sacrificial bonds tend to drive a progressive increase in the coefficient of friction as fracture debris accumulates at the sliding interface. Conversely, strategies employing reversible dynamic bonds, while capable of partially mitigating wear through self-healing, still fail to suppress the rise in friction under high loads and low sliding speeds. Moreover, the more effective the toughening mechanism, the more it tends to “contaminate” the interface through its energy dissipation processes. Elastic deformation is tightly coupled with the lubrication regime: thinner DN gels rapidly enter an ultra-low friction state because geometric confinement suppresses radial expansion and promotes fluid load support, whereas thicker gels undergo slower deformation relaxation and require extended running-in distances to reach steady-state friction. Maintaining a surface hydration layer is a prerequisite for low friction; whether the trigger is debris contamination or an enlarged contact area arising from increased modulus, all failure pathways ultimately converge on breakdown of that hydration layer. Systems capable of rapidly restoring the hydration layer after sliding display markedly superior long-term friction stability compared with those having limited recovery. These phenomena collectively reveal a profound contradiction: in homogeneous networks, the dissipative structures essential for toughening inevitably disrupt the hydrated interface critical for lubrication. Overcoming this toughness–lubrication conflict therefore demands a fundamental shift in design philosophy.

## 3. Reconciling Bulk Mechanical Response and Interfacial Tribology of Hydrogels

Designing hydrogels for load-bearing sliding interfaces demands concurrent optimization of mechanical robustness and interfacial lubricity. Yet these properties arise from fundamentally distinct physical mechanisms and frequently impose conflicting requirements on the network architecture. To address this complexity, this chapter resolves the design problem into three hierarchical performance dimensions: (i) quasi-static bulk mechanics, encompassing ultimate strength and fracture energy; (ii) dynamic cyclic bulk durability, characterized by fatigue threshold and mechanical hysteresis; and (iii) interfacial dynamic behavior, defined by boundary lubrication efficiency and wear resistance.

### 3.1. Quasi-Static Bulk Mechanics: Ultimate Strength and Fracture Energy

Quasi-static mechanical properties underpin load-bearing applications. Their assessment hinges on two distinct metrics: ultimate strength—the maximum tensile or compressive stress a material can sustain before fracture—and fracture energy, defined as the work required to propagate a crack per unit area ($G_{c}$, J/m^2^). Critically, a hydrogel can exhibit high fracture energy through extensive chain stretching and large deformation, yet possess only moderate tensile strength. This section therefore explicitly distinguishes these two parameters when evaluating toughening strategies.

#### 3.1.1. Synergistic Energy Dissipation in Double-Network and Sacrificial-Bond Systems

The double-network (DN) concept pioneered by Gong et al. [59] established the paradigm of sacrificial bond dissipation. In DN gels, the brittle first network fractures ahead of the crack tip into numerous microscopic clusters, dissipating energy over a large process zone [51]. This mechanism elevates the fracture energy to over 1000 J/m^2^—comparable to rubber—while the ultimate compressive strength reaches ~10–20 MPa. Critically, the ultimate tensile strength of DN gels typically remains modest (generally <5 MPa), because the rigid first network restricts chain extensibility.

**Figure 3:**
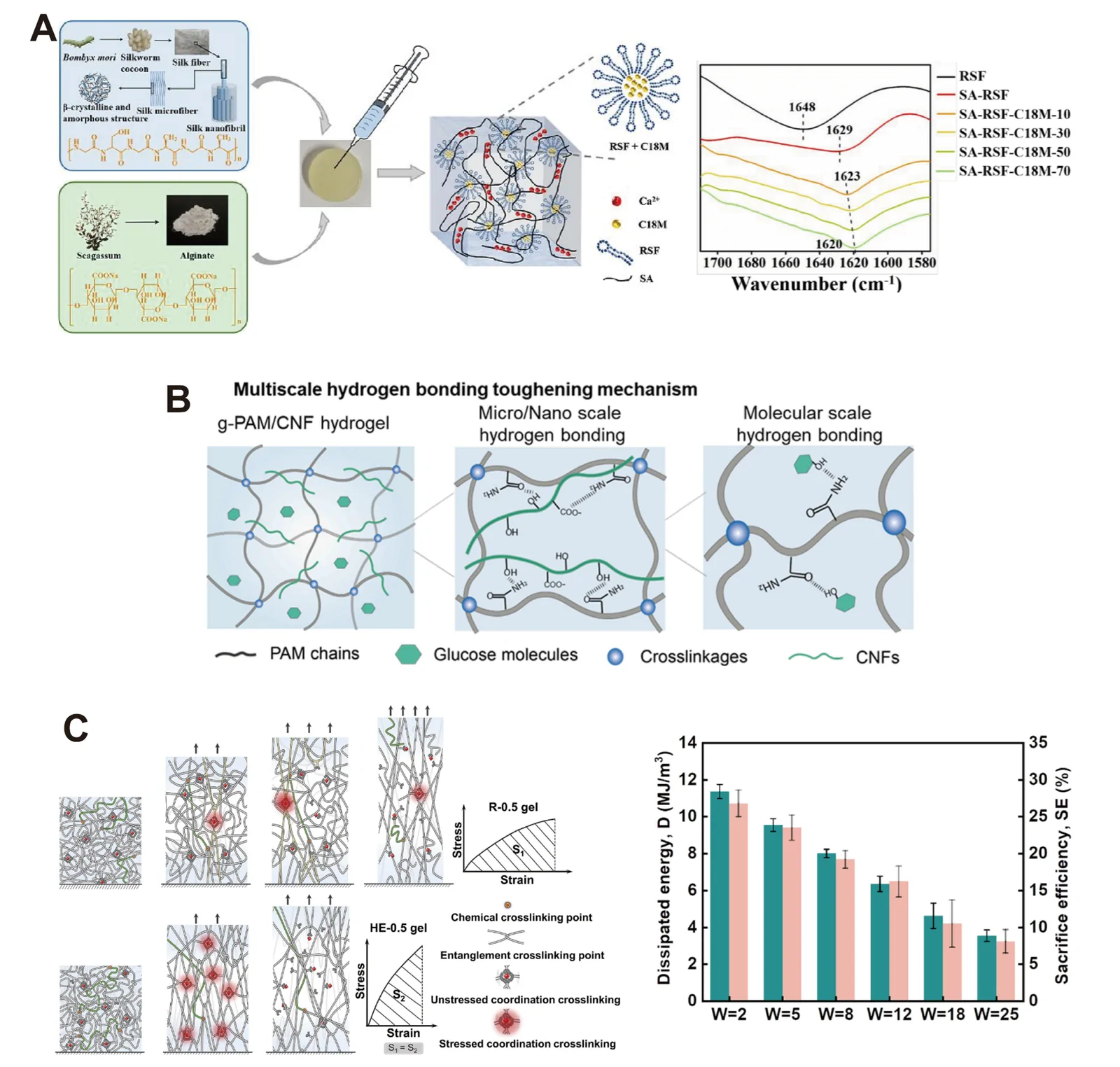
Sacrificial network architectures for toughening. (**A**) Fully physical DN (ionic + hydrophobic crosslinks). Reproduced with permission [61]. Copyright 2020, American Chemical Society. (**B**) Multiscale H-bonding (glucose + CNF): fracture work increased 10.2-fold. Reproduced with permission [62]. Copyright 2025, Royal Society of Chemistry. (**C**) Sacrificial efficiency: entangled network raises efficiency to 26.8%, strength 10.2 MPa, toughness 42.6 MJ/m^3^. Reproduced with permission [63]. Copyright 2025, Elsevier.

This sacrificial principle has been extended to fully physical systems. Sun et al. [60] coupled ionic alginate–Ca^2+^ crosslinking with a covalent PAAm network, achieving notch-insensitive super-stretchability. The continuous dissociation and re-association of ionic bonds during deformation generates extensive internal friction, elevating the fracture energy to approximately 7350 J/m^2^. Similarly, Meng et al. [61] constructed a fully physical DN gel combining ionic crosslinks and hydrophobic association crosslinks (Figure 3A). The hydrophobic microdomains dissociate preferentially under load, substantially enhancing compressive strength. Ye et al. [62] introduced glucose and cellulose nanofibrils (CNF) into a polyacrylamide (PAM) network, constructing a multiscale hierarchical hydrogen-bonding system (Figure 3B). The abundant hydroxyl groups of glucose form dynamic hydrogen bonds with the polymer chains; these bonds rupture preferentially during stretching, dissipating energy, while the CNF nanofibers offset the modulus reduction caused by glucose incorporation and further reinforce the network through topological entanglements. This design synergistically increased the fracture work by a factor of 10.2 relative to the unmodified network. Such multiscale hydrogen-bonding strategies have proven universally applicable—as demonstrated in poly(vinyl alcohol) and gelatin–polyacrylamide systems—revealing a vast design space for hydrogen-bond-based sacrificial networks. A pivotal advance is the introduction of the “sacrificial efficiency” concept by Zhu et al. [63], defined as the ratio of the energy dissipated by sacrificial bond rupture to the total toughness of the material per unit strain (Figure 3C). In networks dominated by conventional chemical crosslinks, sacrificial bonds tend to rupture sequentially with increasing strain, yielding a prolonged, inefficient energy dissipation history. In highly entangled networks, by contrast, dense chain entanglements can slide under stress and redistribute the load, forcing numerous sacrificial bonds to bear stress and rupture nearly synchronously within a narrow strain window—a “collective sacrifice” mode that markedly enhances sacrificial efficiency. A highly entangled hydrogel prepared following this strategy exhibited a tensile strength of 10.2 MPa and a fracture toughness of 42.6 MJ/m^3^; compared with the entanglement-free control gel (strength 4.3 MPa, toughness 3.9 MJ/m^3^), both strength and toughness were substantially improved, and the sacrificial efficiency increased dramatically from 8.1% to 26.8%. The sacrificial efficiency concept thus offers a new theoretical framework and quantitative design criterion for simultaneously overcoming the bottlenecks of hydrogel strength, modulus, and toughness.

From an energy landscape perspective, these designs share a unifying principle: the progressive rupture of sacrificial bonds transforms the localized energy concentration at the crack tip into a broad dissipation zone. The apparent toughness is thus governed by the size of this zone and its volumetric energy dissipation density. Consequently, three primary levers dictate the toughening efficiency of this class of materials: increasing sacrificial bond density, enhancing bond strength, and preserving second-network integrity.

#### 3.1.2. Microphase Separation and Nanocomposite Toughening

Heterogeneous structures offer an alternative quasi-static toughening pathway: internal interfaces dissipate additional energy during deformation through interfacial debonding, cavitation, or particle desorption. In poly(vinyl alcohol) freeze–thaw gels, microcrystalline domains serve as both physical crosslinks and dissipation centers, absorbing substantial energy through their deformation and fragmentation [64]. In nanocomposite hydrogels, clay nanoplatelets [65] or functionalized nanoparticles [66] function as giant crosslinkers and stress-transfer centers, activating dissipative modes such as particle desorption and interfacial slippage upon damage. Unlike sacrificial bond networks, which enhance fracture energy primarily through volumetric dissipation, microphase-separated structures can simultaneously elevate ultimate strength by introducing rigid domains.

**Figure 4:**
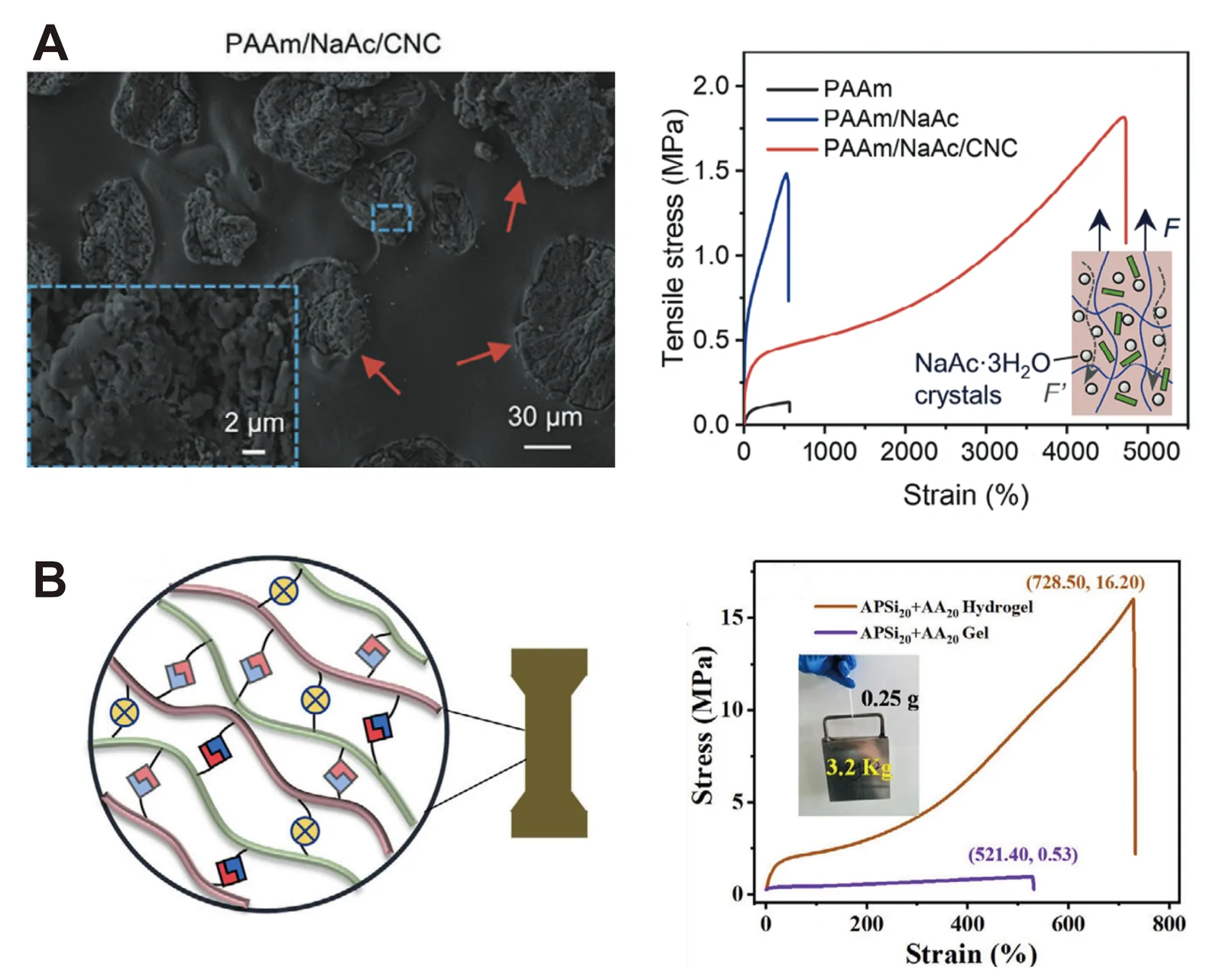
Heterogeneous phase architectures. (**A**) “Island-like” microphase-separated structure induced by competitive hydration of cellulose nanocrystals (CNC). Reproduced with permission [67]. Copyright 2025, Wiley. (**B**) Hydrophobic microdomains and an electrostatic crosslinking network formed via polymerization-induced microphase separation. Reproduced with permission [68]. Copyright 2025, Wiley.

Wang et al. [67] exploited the strong hydration of cellulose nanocrystals (CNC) to competitively disrupt the continuous crystalline phase formed by supersaturated sodium acetate (NaAc) within a polyacrylamide (PAAm) network, transforming it into partially dissolved “island-like” structures (Figure 4A). This treatment reduced the crystallinity from 49.7% to 30.5%, yet counterintuitively produced a comprehensive enhancement of mechanical properties: the optimized hydrogel exhibited a tensile strength of 1.8 MPa, a fracture strain of 4730%, a fracture toughness of 43.1 MJ/m^3^, a fracture energy of 75.4 kJ/m^2^, and a fatigue threshold as high as 3884.7 J/m^2^. The key insight is that “alleviating” rather than “intensifying” microphase separation reconciles ultrahigh extensibility with excellent fracture energy—a concept that breaks from conventional reinforcement strategies which sacrifice ductility for strength, thereby offering a new design paradigm for simultaneously achieving high strength, high toughness, and high fatigue resistance. Zhang et al. [68] adopted a polymerization-induced microphase separation strategy in a system of amino-modified polysiloxane (APSi) and acrylic acid (AA) to construct a multiscale stable network composed of strong and weak electrostatic crosslinks together with hydrophobic microdomains (Figure 4B). This architecture enabled the optimized hydrogel to reach a tensile strength of 16.2 MPa and a toughness of 49.59 MJ/m^3^, outperforming the vast majority of conventional hydrogels and reported silicone hydrogels. The exceptional performance stems from a dual mechanism: rigid hydrophobic microdomains serve as permanent crosslinking points that impart high strength and stiffness, while reversible weak electrostatic interactions dissipate energy efficiently during deformation through dynamic dissociation–reassociation. The synergy of these two mechanisms delivers a simultaneous enhancement of strength and toughness. This work demonstrates how microphase-separated structures can independently regulate two critical quasi-static parameters—strength and fracture toughness—via the dual strategy of “rigid reinforcement” and “reversible sacrifice,” providing an important theoretical foundation for the design of high-performance hydrogels. In addition, the hydrogel exhibits excellent self-healing, anti-swelling behavior, and water-triggered shape memory.

#### 3.1.3. Topological Toughening and Disentanglement

**Figure 5:**
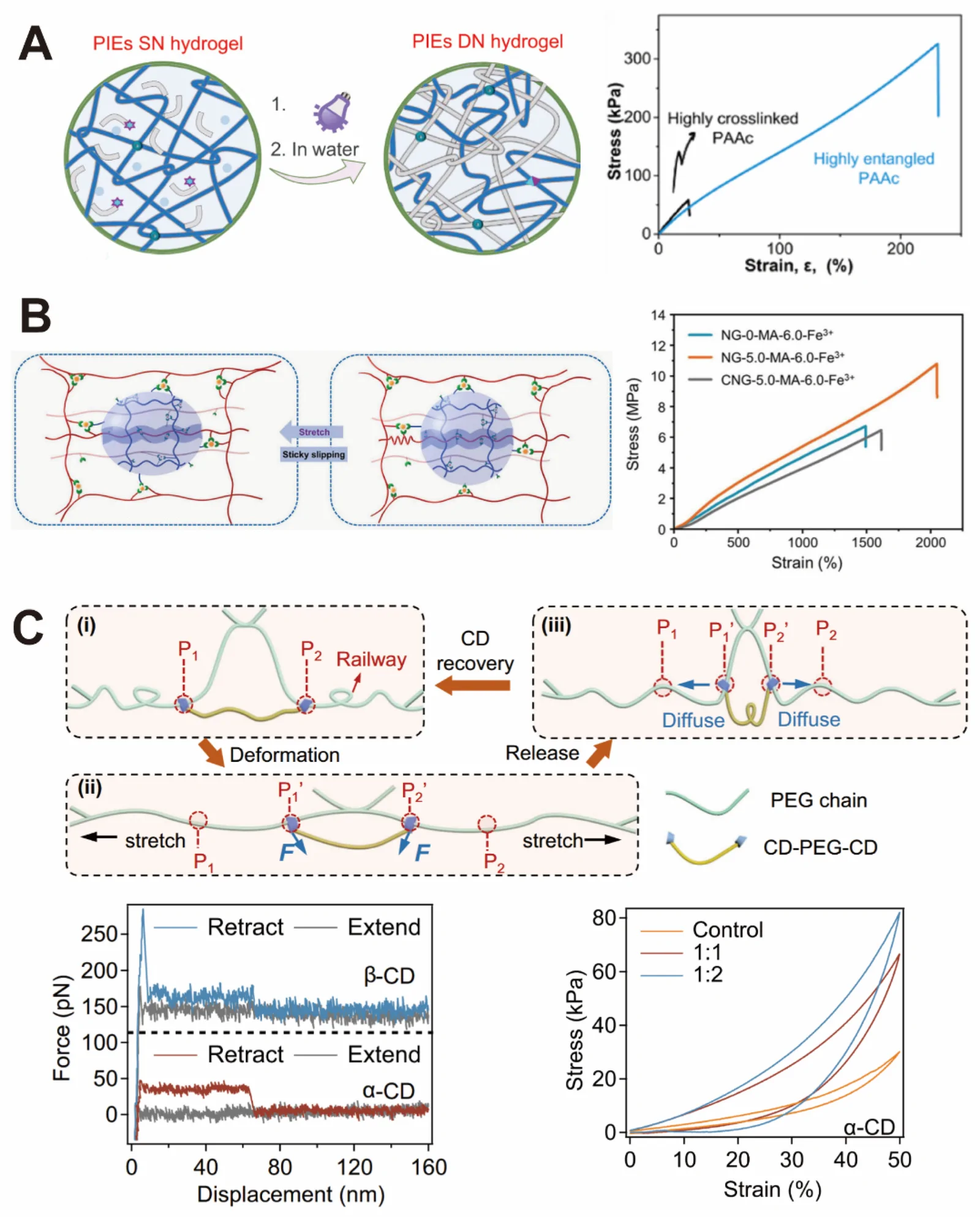
Topological architectures for low-hysteresis toughening. (**A**) Polymerization-induced entanglement (PIE) double-network hydrogel composed of a fabric-like first network dominated by physical entanglements and a second network with enhanced chain entanglements. Reproduced with permission [69]. Copyright 2025, American Chemical Society. (**B**) Nano-domain reconfiguration hydrogel consisting of a Fe^3+^-coordinated nanogel network interpenetrated with linear PAMA chains that undergo sticky sliding under small deformation and nanogel rupture under large strain. Reproduced with permission [70]. Copyright 2022, Elsevier. (**C**) Molecular-friction damping hydrogel built from a CD-PEG-CD “chain walker” threaded into a covalently crosslinked four-arm PEG track network, dissipating energy without covalent bond scission. (i) Initial state: the chain walkers reside on the relaxed PEG tracks, whose ends are fixed. (ii) Loading (stretching) state: external force stretches the PEG tracks, permitting the chain walkers to slide along them (P_1_→P_1_′, P_2_→P_2_′); this sliding dissipates mechanical energy through intermolecular friction—arising, for example, from dynamic hydrogen-bonding interactions—between the cyclodextrin rings and the PEG chains. (iii) Recovery (unloading) state: upon load removal, the covalently crosslinked PEG tracks rapidly recoil elastically; the chain walkers, no longer tethered, quickly return to their initial equilibrium positions, allowing the hydrogel to restore its damping capacity within seconds. Reproduced with permission [72]. Copyright 2024, Springer Nature.

Topological structures offer a distinct toughening mechanism: chain entanglements act as “slidable crosslinks” that redistribute stress without irreversible bond rupture. Zhang et al. [69] fabricated polymerization-induced entanglement (PIE) double-network hydrogels (Figure 5A) in which the first network, dominated by physical entanglements, adopts a “fabric-like” topology. Under stretching, the sliding of entanglement points permits segmental reorientation, uniformly distributes stress, and delays localized fracture, thereby markedly reducing hysteresis while preserving high toughness. The optimized PIE hydrogel achieved a tensile strength of 1.25 MPa, a fracture toughness of 5800 J/ m^2^, and exceptionally low hysteresis (below 0.10 at 225% strain, stabilizing at ~0.15 after 10 cycles), exhibiting elastomer-like mechanical behavior.

Yuan et al. [70] extended topological dissipation to multiscale systems via a nanodomain remodeling strategy (Figure 5B): linear poly(acrylamide-co-methacrylic acid) (PAMA) chains interpenetrate a nanogel network crosslinked by Fe^3+^ coordination, constructing a multilevel energy dissipation system. Under small deformation, the interpenetrating chains undergo “sticky sliding” within the nanogels, dissipating energy through repeated “catch-and-release” of coordination bonds; under large strain, the nanogels themselves rupture and reorganize, synergistically dissipating further energy and redistributing stress. This synergistic multi-mechanism approach enabled the hydrogel to attain a tensile strength of 10.8 MPa, a fracture strain of 2048%, and a toughness of 111.8 MJ/m^3^. In topological networks, crosslinks slide freely along polymer chains—much like a pulley system—automatically equalizing chain tension, delaying stress concentration, and shifting dissipation from chain scission to viscous sliding and chain disentanglement [71]. Because this toughening relies on topological rearrangement rather than irreversible bond rupture, it confers superior recoverability under repeated bulk loading [69,70]. This low-hysteresis, highly reversible behavior is ideally suited to the mechanical demands of surface layers in sliding contacts, where cumulative viscous dissipation and network damage under cyclic shear would otherwise accelerate friction buildup and wear. Although the direct manifestation of these topological sliding mechanisms under interfacial contact conditions—with their multiaxial stress states and characteristic boundary shear rates—has yet to be experimentally demonstrated, the bulk findings already provide a compelling design rationale: surface layers engineered with analogous reversible energy-dissipation pathways are, in principle, better suited to sustain lubrication stability over prolonged cycling.

### 3.2. Dynamic Cyclic Bulk Durability: The Intrinsic Conflict Between Fatigue Resistance and Mechanical Hysteresis

Quasi-static toughness governs resistance to sudden overload, but long-term service under cyclic loading—as in joint motion or device operation—demands fatigue resistance, quantified by the fatigue threshold $G_{0}$, the critical energy release rate below which a crack does not propagate. Crucially, fatigue resistance does not correlate positively with quasi-static fracture energy. The large mechanical hysteresis that often enhances quasi-static toughness is, however, intrinsically detrimental to fatigue durability when it arises from irreversible sacrificial mechanisms such as covalent bond scission or chain pull-out. From an energy-dissipation perspective, pronounced hysteresis under cyclic loading entails significant interfacial heat generation that is expected to compromise surface hydration-layer stability; yet the critical temperature threshold for such destabilization, and the extent to which thermal effects compete with mechanical wear or fluid-film rupture in sliding hydrogel contacts, remain to be quantified.

Classic DN hydrogels, despite their high quasi-static fracture energy, exhibit relatively low fatigue thresholds (typically $G_{0}<50\;{J/m}^{2}$) because the brittle first network fractures irreversibly in each loading cycle. The resulting debris accumulates as microstructural damage, accelerating crack propagation under cyclic loading. This fundamental limitation arises because the fatigue threshold is governed by the minimum energy required for a single bond rupture, whereas the quasi-static fracture energy integrates the cumulative dissipation from numerous sacrificial bond ruptures.

Systems optimized for high quasi-static dissipation, however, inevitably incur a hysteresis penalty. Xu et al. [72] developed a damping hydrogel based on molecular sliding friction that achieves rapid energy dissipation and recovery within seconds (Figure 5C). A “chain walker” (CD-PEG-CD), composed of two cyclodextrin (CD) units connected by a short polyethylene glycol (PEG) spacer, was threaded into a covalently crosslinked four-arm PEG “track” network. Because energy dissipation proceeds without covalent bond scission, the PEG-α-CD hydrogel retained over 94% of its maximum stress and dissipated energy after continuous compression cycles without rest intervals, demonstrating fatigue resistance difficult to attain with conventional viscoelastic dissipation systems. This trade-off is intrinsic: effective fatigue resistance demands reversible, low-energy-loss pathways, whereas quasi-static toughness relies on high-energy, irreversible sacrificial bond rupture.

Fatigue resistance and quasi-static fracture energy are thus distinct and often mutually antagonistic parameters. High hysteresis benefits quasi-static toughness but compromises fatigue durability and can undermine surface lubrication through kinetic constraints—specifically, slow viscoelastic relaxation of the network impedes interfacial rehydration between sliding cycles [73,74]—while the additional contribution of hysteresis-induced frictional heating to hydration-layer destabilization remains a conjecture that awaits direct interfacial thermometry under sliding contact. Therefore, designing durable, low-friction hydrogels requires treating low hysteresis and high fatigue threshold as independent design objectives, rather than assuming that high fracture energy guarantees long-term reliability.

### 3.3. Interfacial Dynamic Behavior: Boundary Lubrication and Wear Resistance

The tribological performance of hydrogels at sliding interfaces is governed by two independent metrics: the steady-state coefficient of friction (COF), which reflects the interfacial shear stress under lubrication, and the specific wear rate (volume loss per unit sliding distance and load), which quantifies material removal. Notably, a low COF does not guarantee low wear, and vice versa. Hydrogel friction occurs almost exclusively in aqueous environments, where lubrication behavior is dictated by a combination of bulk hydrodynamic effects, elastohydrodynamic surface deformation, and the properties of interfacial hydration layers. A comprehensive understanding of this multi-level lubrication is a prerequisite for designing low-friction surfaces. Based on the dominant energy dissipation pathway, hydrogel lubrication can be classified into two fundamental modes: fluid-film lubrication and boundary lubrication [75]. In fluid-film lubrication, the load is sustained by viscous shear of the intervening fluid film; in boundary lubrication, the surface physicochemical properties directly dictate the shear strength of the solid–solid contact region.

#### 3.3.1. Stribeck Curve Mapping of Lubrication Regimes

The classical Stribeck curve partitions friction into three regimes according to the operating parameters: boundary, mixed, and hydrodynamic (full-film) lubrication. For soft, porous hydrogels, the transitions between these regimes are further modulated by osmotic pressure and network deformation. At low sliding speeds and high loads, the system operates in the boundary lubrication regime: surface polar groups anchor tightly bound water molecules, and the resulting strong hydration repulsion maintains a nanometer-thick (~1–5 nm) fluid-like layer that suppresses direct solid–solid contact. Lubrication in this regime therefore depends on the low shear strength of this hydration layer and, when present, on adsorbed polymer chains. At higher sliding speeds or lower loads, a localized macroscopic fluid film can form within the contact gap, giving rise to mixed lubrication. When the film thickness becomes sufficient to completely separate the opposing surfaces—comparable to or moderately exceeding the composite surface roughness—the system approaches full-film elastohydrodynamic lubrication (EHL), in which the load is entirely supported by the hydrodynamic pressure generated by the entrained external fluid. It is precisely this self-regulating, motion-adaptive lubrication mechanism that natural articular cartilage exploits to maintain extremely low friction over a wide range of operating conditions [76].

However, the dynamic behavior of polymer chains and the intrinsic poroelasticity of hydrogels frequently inhibit the formation of full-film lubrication. Shoaib et al. [77] employed colloidal-probe atomic force microscopy to measure friction between polyacrylamide hydrogels of varying moduli and silica spheres. They found that the friction force first decreased and then increased with sliding speed, with the transition speed correlating with the elastic modulus. In the low-speed regime, lubrication is governed by polymer-chain adsorption–desorption kinetics: lower-modulus hydrogels, owing to their compliant networks and high segmental re-adsorption activity, sustain a more intact interfacial film and thus achieve lower friction. In the high-speed regime, however, adsorption probability becomes the dominant factor, and the correlation between the rate of friction increase and modulus weakens. Stick–slip analysis further revealed that direct chain–probe contact persists even at high velocities, and the system never enters the full-film fluid-lubrication regime; stick–slip is more pronounced under higher loads and lower speeds, consistent with the highest adhesion energy observed for hydrogels of intermediate crosslinking density. This study highlights the critical role of polymer-chain fluctuation dynamics in boundary lubrication at hydrogel–solid interfaces. Hydrogel designers must recognize that a hydrodynamic effect derived solely from increasing the viscosity of the external lubricating fluid is inherently fragile; it is boundary hydration lubrication that ultimately governs the lower limit of friction and the start-up performance.

#### 3.3.2. Surface Hydration Layers and Molecular Mechanisms of Boundary Lubrication

**Figure 6:**
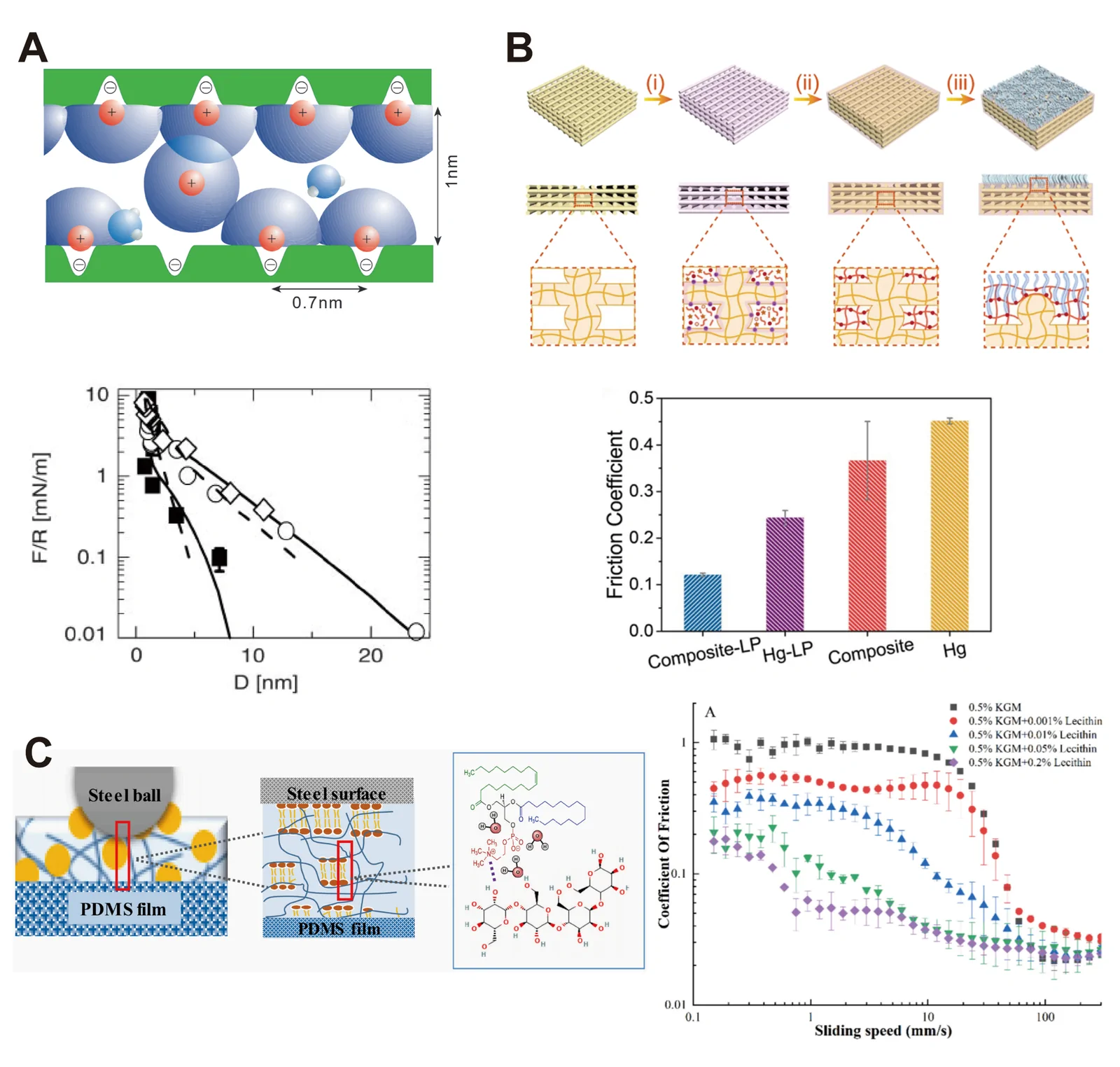
Hydration lubrication. (**A**) Mechanism of hydration lubrication.When the fluid film is compressed to approximately 1.0 nm, bound water molecules retain bulk-water-like shear fluidity. In concentrated salt solutions, hydrated Na^+^ ions promote the formation of a highly ordered interfacial hydrogen-bond network via a rapid exchange mechanism. This hydration layer sustains normal pressures of tens of megapascals without rupture, yet its shear strength remains comparable to that of a dilute solution, giving rise to fluid-like low-friction behavior. Reproduced with permission [78]. Copyright 2002, AAAS. (**B**) Thick poly(sulfopropyl methacrylate) (PSPMA) polyelectrolyte brushes as a lubrication layer. (i) Surface activation of the 3D elastomer scaffold via benzophenone soaking; (ii) UV-triggered in situ polymerization and covalent grafting of the polyacrylamide hydrogel matrix onto the scaffold; (iii) grafting of PSPMA polyelectrolyte brushes by SSI-ATRP to form the surface lubrication layer. Reproduced with permission [82]. Copyright 2022, American Chemical Society. (**C**) Effect of lecithin on hydrogel lubrication. Reproduced with permission [84]. Copyright 2024, Elsevier.

Under boundary lubrication, hydrogel friction is governed by the modified Bowden–Tabor model, scaling with the load-supporting area of the interfacial fluid film and the shear strength of the interfacial hydration layer. Accordingly, friction reduction can be pursued through two direct pathways: first, by exploiting high elasticity and low modulus to render the surface sufficiently compliant, such that a complete, continuous interfacial hydration layer forms under load, preventing direct solid-phase contact and allowing friction to be dominated entirely by the low-shear behavior of the hydration layer; and second, by further lowering the shear strength of this hydration layer—for example, through enhanced surface hydrophilicity or the introduction of polymer brushes or zwitterionic groups that strengthen hydration and facilitate shear within the interfacial water layer. Klein et al. [78] used surface force apparatus experiments to demonstrate that charged surfaces can anchor a tightly bound hydration layer only a few nanometers thick (Figure 6A). Owing to its highly structured hydrogen-bond network, this hydration layer generates exceptionally strong repulsive forces, withstanding normal pressures of tens of megapascals without rupture, while its shear strength remains comparable to that of a dilute solution, thereby exhibiting fluid-like low friction. This “hydration lubrication” mechanism has since been widely invoked to explain the superior lubricity of natural articular cartilage, whose surface is rich in negatively charged proteoglycans and lipid layers. Luo et al. [79] developed a multi-physical crosslinking network hydrogel composed of poly(vinyl alcohol), chitosan, and sodium alginate (TPCS). By harnessing the triple synergy of freeze–thaw-induced PVA crystalline domains, hydrogen bonds between chitosan and PVA, and ionic coordination between chitosan amino groups and sodium alginate carboxyl groups, the hydrogel achieved a coefficient of friction (COF) of 0.044. The negatively charged carboxyl groups of sodium alginate effectively thickened the surface hydration lubrication layer, validating the pivotal role of surface charge in enhancing hydrogel lubrication.

The key to achieving hydration lubrication in hydrogel systems is to introduce surface chemical functionalities that firmly anchor the hydration layer. Common molecular designs include sulfonate groups [80], phosphorylcholine groups [75], and polyelectrolyte brushes [81]. For instance, Zhao et al. [82] used a covalently crosslinked polyacrylamide hydrogel composite as a load-bearing substrate and grafted dense polyelectrolyte brushes poly(sulfopropyl methacrylate) (PSPMA) onto its surface via surface-initiated atom transfer radical polymerization (SSI-ATRP) to serve as a lubrication layer (Figure 6B). Under water lubrication, the grafted composite exhibited a COF of ≈0.1, markedly lower than that of the pure hydrogel (0.45) and the ungrafted composite (0.37), with negligible surface wear and stable low friction maintained over a wide range of loads and frequencies. Through strong hydration, these hydrophilic groups form a “quasi-solid water film” that resists squeeze-out, enabling the friction coefficient (μ) to remain as low as 0.01–0.001 even under extremely high contact stress.

Furthermore, the cartilage-mimetic “surface-active phospholipid” strategy has been adapted for hydrogel design. Introducing hydrophobic lipid end-groups or vesicles at the surface can generate a low-shear-strength slip plane in situ at the friction interface [75,83]. Pang et al. [84] systematically examined the effect of lecithin on the lubrication of konjac glucomannan (KGM) hydrogels (Figure 6C). By preparing gels with varying KGM concentrations and lecithin contents, they found that trace amounts of lecithin significantly altered the shear viscosity and storage modulus, reorganized the microscopic network structure, substantially enhanced boundary lubrication, and markedly reduced the COF. Mechanistically, lecithin self-assembles into colloidal particles that interact with KGM via hydrogen bonds and adsorb onto the counterface during sliding, forming a hydration lubrication layer while simultaneously weakening viscous bridging between KGM molecules, thereby endowing the gel with an excellent “adhesive yet slippery” characteristic. Similarly, Feng et al. [85] prepared zwitterionic copolymer hydrogels incorporating phospholipid liposomes (HSPC, including multilamellar vesicles MLV and small unilamellar vesicles SUV) and hyaluronic acid (HA). HA alone failed to improve lubrication; however, the introduction of HSPC significantly reduced the COF, with SUV outperforming MLV owing to their uniform size and structural stability. When HSPC (SUV) was combined with HA, the two components formed a more uniform and robust lipid-based hydration boundary layer via electrostatic interactions. HA promoted the ordered assembly and continuous replenishment of liposomes at the hydrogel interface, preventing them from being squeezed out and thus enhancing the stability and load-bearing capacity of the lubrication layer.

Notably, friction under this hydration lubrication regime is nearly independent of the bulk modulus: the surface friction can be governed by a molecular brush that is decoupled from the bulk network, while the bulk can be dedicated to load-bearing and toughening. This provides a physical basis for realizing a bulk-surface decoupled design.

#### 3.3.3. Rehydration and Wear

The friction of hydrogels is governed by the synergistic interplay of poroelastic biphasic lubrication, boundary lubrication, and molecular-scale network dynamics. As fluid-saturated porous media, hydrogels undergo pressure-driven fluid exudation and deformation-induced rehydration during sliding. Pore fluid redistribution transfers part of the applied load to the pore pressure, thereby reducing the solid-phase contact stress; simultaneously, the shear-induced deformation and orientation of surface polymer chains alter the shear viscosity of the boundary layer. These factors together dictate the rate-dependent, time-dependent, and hysteretic friction characteristics of hydrogels.

**Figure 7:**
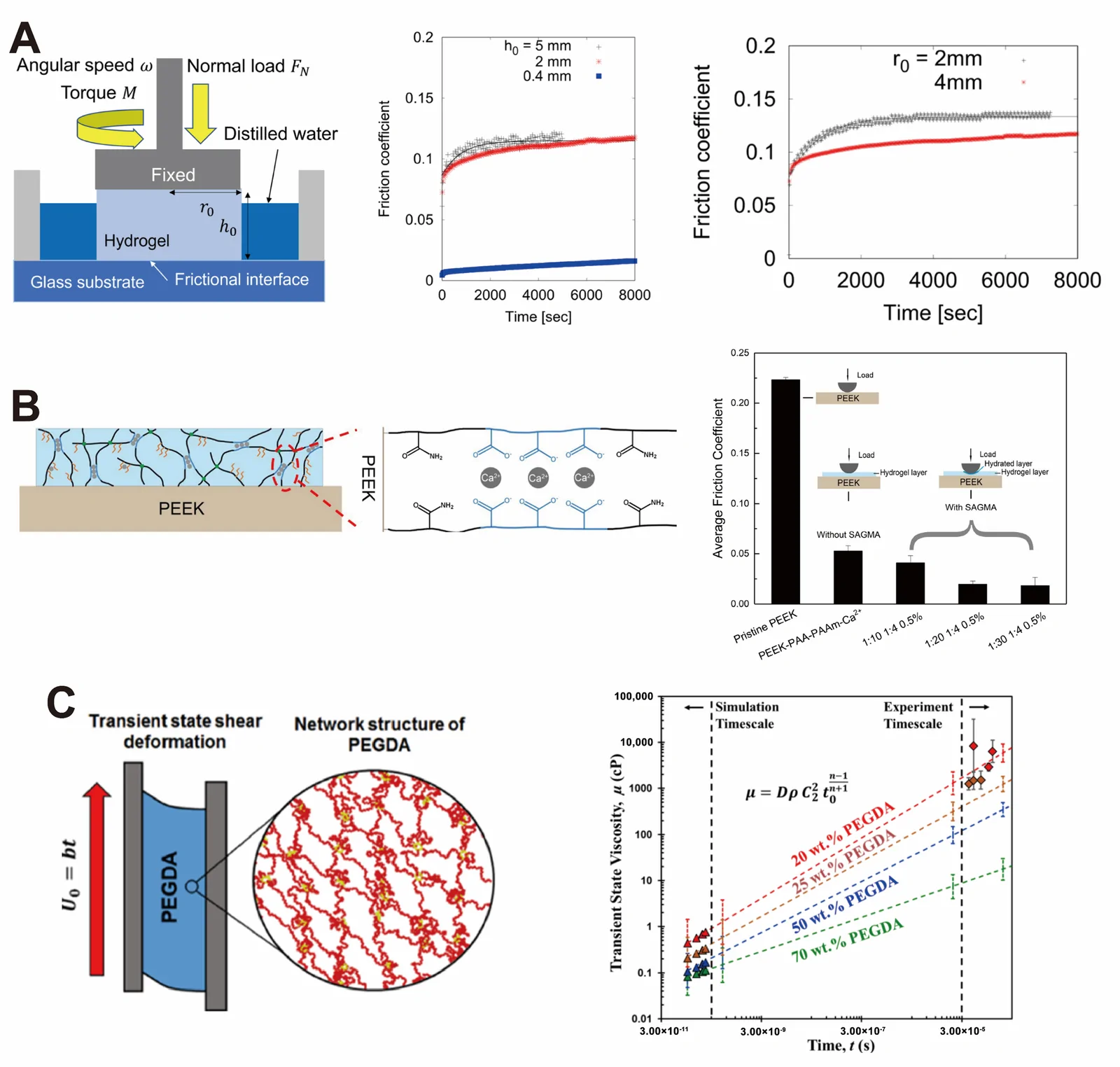
Surface Fluid Dynamics and Rehydration. (**A**) Biphasic lubrication theory: thickness governs fluid load support and COF evolution. Reproduced with permission [73]. Copyright 2025, Springer Nature. (**B**) PEEK–hydrogel–brush composite: rehydration rate dictates COF under reciprocating sliding. Reproduced with permission [74]. Copyright 2020, Elsevier. (**C**) PEGDA transient rheology: shear-thickening linked to two-stage chain deformation, governing boundary-layer viscosity. Reproduced with permission [86]. Copyright 2019, American Chemical Society.

Yamaguchi et al. [73] developed a cylindrical hydrogel friction model under rotational shear based on biphasic lubrication theory, revealing the intrinsic origins of the aforementioned time dependence and sample-size effects (Figure 7A). Under sliding, pore fluid migrates from the center toward the periphery under a pressure gradient, and the resulting hydrostatic pressure sustains part of the normal load, so that the coefficient of friction (COF) gradually rises from a low initial value to a steady state. This relaxation process is governed by the hydrogel radius and thickness; notably, in thin samples, the constraint of the upper surface suppresses radial expansion and substantially enhances fluid load support, causing the initial friction to drop sharply with decreasing thickness. This indicates that geometric design is a key lever for regulating bulk fluid transport to achieve low friction. Liu et al. [74] examined a polyether ether ketone (PEEK)–hydrogel–molecular brush “soft-on-hard” composite and systematically elucidated the synergistic friction-reduction mechanism of biphasic and boundary lubrication (Figure 7B). During reciprocating sliding, the compressed hydrogel network extrudes internal lubricating fluid, providing fluid load support, and after the counterface passes, the network reabsorbs fluid to accomplish rehydration. When the sliding speed exceeds the rehydration rate, inadequate lubricant replenishment increases direct solid-phase contact, causing the COF to rise markedly with pronounced rate dependence; a higher normal load, however, helps sustain effective fluid-film lubrication, while the surface molecular brushes further lower the solid-phase shear viscosity by forming a hydration boundary layer. These findings confirm that maintaining an open surface network to promote rapid rehydration is as crucial as surface chemical regulation for low-friction design. At the molecular scale, Luo et al. [86] employed molecular dynamics simulations and split Hopkinson pressure bar experiments to investigate the transient rheological response of poly(ethylene glycol) diacrylate (PEGDA) hydrogels at high shear rates (Figure 7C). They discovered a distinct non-Newtonian shear-thickening behavior linked to a two-stage network deformation: an initial stage dominated by conformational changes and orientational reorganization of polymer chains, followed by a later stage involving significant stretching of bond lengths and bond angles. In low-concentration hydrogels, the average mesh size expands more rapidly, allowing more chain segments to enter the high-resistance second stage, thereby producing higher transient viscosity and a stronger shear-thickening effect. This dynamic microstructural evolution offers a molecular-level interpretation for transient viscosity enhancement in polymer networks; when extrapolated to the boundary layer of sliding contacts, it suggests that similar chain-reorganization and bond-stretching events may contribute to the effective shear viscosity and momentum diffusion at the interface, thereby providing a potential structural basis for macroscopic friction hysteresis and rate dependence. Such cross-scale extrapolation from bulk impact rheology to interfacial shear, while physically plausible, awaits direct experimental validation under boundary-lubrication conditions.

Collectively, the friction process of hydrogels involves pressure-driven fluid exudation and deformation-induced rehydration; the redistribution of pore fluid transfers part of the load to the pore pressure, mitigating solid-phase contact stress [87,88], while the shear-induced deformation and orientation of surface chains modify the boundary-layer viscosity. The coupling of these cross-scale mechanisms governs the complex time-dependent friction response. Consequently, low-friction hydrogel design must holistically integrate three critical elements: (i) geometric constraints for bulk fluid transport, (ii) an open surface network that permits rapid rehydration, and (iii) a stable hydration boundary layer constructed through surface chemical modification. This idealized design framework, however, confronts a fundamental materials science dilemma: toughening strategies—such as increasing crosslinking density or incorporating fillers—while enhancing mechanical properties, intrinsically reduce water permeability, impede rehydration, and exacerbate wear. Nanocomposite hydrogels exemplify this trade-off. Arjmandi et al. [53] found that silica nanoparticles reduced wear volume by 69% under bovine serum lubrication; yet under dry sliding conditions, a high nanoparticle concentration (4 wt%) actually increased wear. This reveals that the tribological benefits of nanofillers are strongly lubrication-dependent: under boundary lubrication, the exposure of abrasive particles can exacerbate wear, irrespective of how low the coefficient of friction may be.

Under boundary lubrication, the coefficient of friction is primarily governed by surface chemistry and hydration-layer stability, which set the lower limit of interfacial shear stress. However, in homogeneous networks, bulk mechanics—including elastic modulus, network permeability, and poroelastic response—exert substantial indirect control over friction through contact-area evolution, fluid-film formation, and rehydration kinetics. This inherent coupling means that optimizing surface hydration alone is insufficient: a hydrogel can exhibit an ultra-low coefficient of friction, yet if rehydration is sluggish—owing to a dense network—or if rigid fillers are exposed at the interface, rapid wear and friction escalation can still ensue. Therefore, within homogeneous network designs, simultaneous optimization of surface hydration (for a low COF) and bulk porosity/rehydration kinetics (for low wear) remains necessary, but these requirements are often mutually conflicting.

### 3.4. Synergy of Toughness and Low Friction Through Functional Decoupling

The preceding discussion exposes several fundamental incompatibilities inherent in homogeneous networks: quasi-static toughness demands high-density sacrificial bonds or rigid phases, yet these increase hysteresis and reduce permeability; fatigue resistance requires low hysteresis and reversible, low-energy dissipation pathways, which conflict with high quasi-static fracture energy; a low coefficient of friction necessitates a soft, highly hydrated, and permeable surface layer; and low wear demands rapid rehydration and the avoidance of abrasive rigid-phase exposure at the sliding interface. The only systematic solution to this multidimensional conflict is spatial functional decoupling—partitioning bulk dissipation and surface lubrication into distinct structural domains within the same material. This functional decoupling principle is already embodied in natural articular cartilage: the superficial zone (~10–20% of the thickness), dominated by collagen fibrils oriented parallel to the surface and lubricating proteins, imparts low friction, whereas the deep zone, characterized by fibers perpendicular to the surface and densely packed proteoglycans, provides load-bearing capacity [89]. Decoupling hinges on constructing spatially asymmetric or gradient architectures. In an idealized configuration, the bulk consists of a highly dissipative network—such as a dense double-network or nanocomposite—that delivers load-bearing capacity and toughness, whereas the surface is a soft, highly hydrated, and conformable lubrication layer—for instance, polymer brushes [81] or a porous hydration layer [90]—that readily complies with the counterface. This physical separation permits a propagating crack tip to fully engage the bulk dissipation mechanisms, while the sliding surface sustains a stable hydration film within the contact zone. Because the shear stress under hydration lubrication is exceptionally low, the frictional work dissipated within the surface layer contributes minimally to fatigue and damage of the bulk, effectively decoupling the mechanical and tribological properties [91,92]. A robust decoupling design must satisfy three criteria: (1) Spatial separation—the dissipation zone must reside within the bulk at a distance from the surface equivalent to at least several times the process-zone size, and the surface layer must avoid high-density sacrificial bonds and rigid heterogeneous phases. (2) Interfacial compatibility—a robust chemical or physical bond between the bulk and the surface layer is essential to prevent delamination during sliding. (3) Hydration pathways—the surface layer must retain sufficient porosity or hydrophilic channels to enable rapid water transport from the bulk to the contact interface during deformation.

#### 3.4.1. Vertically Gradient Network Design

**Figure 8:**
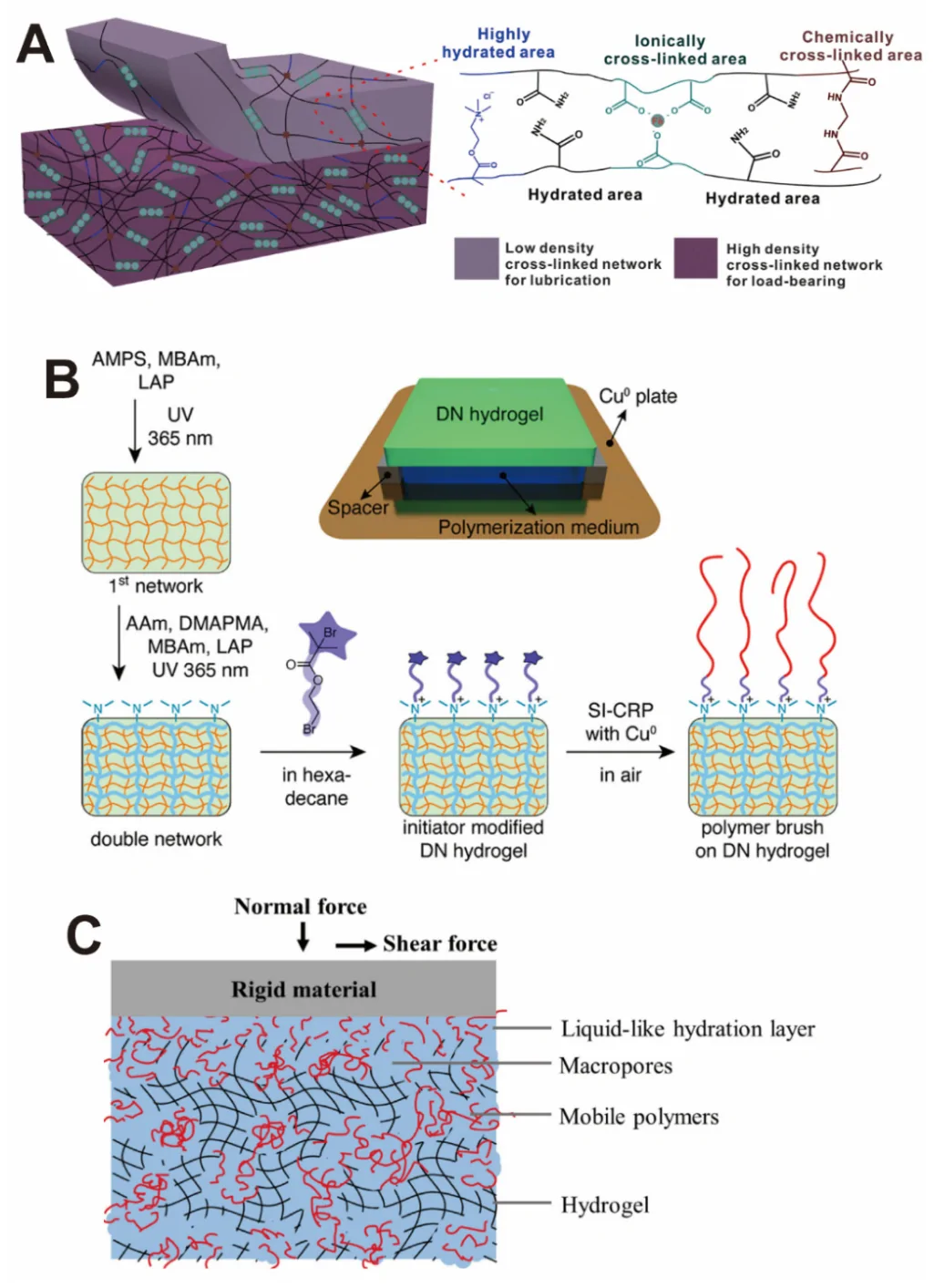
Tough, low-friction hydrogel designs. (**A**) Vertical gradient: porous surface for lubrication, dense bulk for load-bearing. Reproduced with permission [93]. Copyright 2016, American Chemical Society. (**B**) Bilayer: DN bulk + grafted brushes. Reproduced with permission [95]. Copyright 2021, Elsevier. (**C**) Porous scaffold infiltration: mobile chains provide sustained lubrication; scaffold isolated from friction interface. Reproduced with permission [90]. Copyright 2020, Elsevier.

Vertically gradient network design centers on controlling polymerization conditions to generate a continuous or quasi-continuous gradient in crosslinking density or network architecture along the thickness direction. This design is motivated by the exquisite sensitivity of hydrogel friction to surface-layer structure: a sparse surface network promotes hydration-layer formation and lowers interfacial shear resistance, whereas a dense bulk network provides load-bearing capacity and deformation resistance. Lin et al. [93] leveraged oxygen inhibition at hydrophobic mold surfaces to fabricate a one-step gradient structure. Oxygen quenching of radicals at the surface yields extremely low crosslinking and a loose, porous morphology, while the oxygen-shielded bulk polymerizes into a dense network, further reinforced by Fe^3+^ secondary crosslinking in the bottom region. This gradient architecture achieves functional differentiation: a thin, soft, water-rich top layer provides lubrication, while a dense, stiff bottom layer bears the load (Figure 8A); their synergy imparts both ultra-low friction and high load-bearing capacity. The key advantage of this approach is one-step fabrication—the gradient structure is realized simply by tuning mold surface chemistry, without subsequent grafting or assembly steps. Gradient structures can also be generated photochemically. Gu et al. [94] used long-wave ultraviolet light to reduce Fe^3+^, constructing a gradient network in which crosslinking density increases progressively from the surface inward, along with a porous surface. They then introduced a superhydrophilic comb-like polymer, analogous to aggrecan, into the pores and firmly anchored it via catechol–Fe^3+^ coordination. The resulting material sustained a compressive modulus of ~15 MPa and an ultra-low coefficient of friction (COF) of ≈0.0028 over 36,000 cycles under a 20 N load, while maintaining stable lubrication and excellent wear resistance under variable loads and surface-damaged conditions—thereby validating the long-term reliability of vertically gradient network designs.

#### 3.4.2. Bilayer Heterogeneous Structures

In contrast to the continuous gradient transition in vertically gradient networks, bilayer heterogeneous structures feature a well-defined interface: the bulk is a high-strength hydrogel network, while the surface is a grafted polymer brush layer whose chemical composition and architecture are independent of the bulk. This pathway permits truly independent optimization—the bulk can be selected from any tough hydrogel system (e.g., DN, nanocomposite, highly entangled networks), while the surface brush chemistry, grafting density, and chain length are tailored exclusively to lubrication requirements, free from the constraints of bulk crosslinking chemistry. For instance, Rong et al. [96] used atom transfer radical polymerization (ATRP) to graft anionic polyelectrolyte and zwitterionic polymer brushes onto DN hydrogel surfaces. The soft outer layer provides effective hydration lubrication, while the tough substrate bears the load, unifying high lubricity and high load-bearing capacity and sustaining low friction even under high contact pressure. Zhang et al. [95] adopted a different surface-initiated approach, SI-Cu^0^CRP (copper plate-mediated surface-initiated controlled radical polymerization) (Figure 8B), to graft polymer brushes onto DN hydrogels at ambient temperature. They first introduced dimethylaminopropyl methacrylamide (DMAPMA) monomer into the DN network as a reactive site and then grew poly(2-methacryloyloxyethyl trimethylammonium chloride) (PMETAC) brushes via SI-Cu^0^CRP. The resulting hydrogel achieved an ultra-low COF of 0.001–0.004 under water lubrication, rivaling the friction performance of synovial joints. This method combines ambient-temperature processing with broad monomer versatility, enabling the grafting of polymer brushes with a wide range of chemical compositions onto DN hydrogel surfaces.

#### 3.4.3. Porous Scaffold Infiltration

In contrast to surface-layer strategies such as vertically gradient networks and bilayer heterogeneous structures, porous scaffold infiltration operates from within the bulk: a macroporous hydrogel scaffold is first prepared, and mobile polymer chains are subsequently infiltrated into the pores. The scaffold provides mechanical support and compression resistance, while the infiltrated chains serve as a supplementary hydration lubrication source at the friction surface, dissipating shear energy through their mobility without compromising the scaffold. Mu et al. [90] pioneered this approach by fabricating a macroporous polyacrylamide scaffold via freeze–thaw cycling and then immersing it in a solution of mobile polymer chains, which diffused into the pores (Figure 8C). Compared with a neat hydrogel or a water-filled porous hydrogel, the polymer-infused macroporous hydrogel exhibited significantly lower friction and sustained low friction over prolonged sliding. This design mimics the division of labor in synovial joints: the macroporous scaffold stores and transports the lubricating medium, while the mobile polymer chains provide hydration lubrication under shear. The key advantage of this pathway is that the reinforcing scaffold remains physically isolated from the friction interface. In typical nanocomposite hydrogels, rigid nanofillers are dispersed throughout the network; excessive loading or non-uniform dispersion exposes hard particles at the surface, which then act as abrasives and exacerbate wear. In the porous scaffold infiltration design, by contrast, the reinforcing scaffold is confined to the bulk and never directly contacts the counterface—the friction interface is composed solely of the hydrated polymer-infiltrated layer and the lubricating pore fluid. This configuration fundamentally circumvents the reinforcement–wear coupling inherent in nanocomposite systems.

To provide a consolidated overview of the distinct design philosophies discussed above, (Table 1) qualitatively compares the key advantages, limitations, and structural principles of the major hydrogel systems. For detailed quantitative data under specific test conditions, the reader is referred to Supplementary Table S1.

## 4. Applications of Tough, Low-Friction Hydrogels

With their high water content, excellent biocompatibility, and tissue-like mechanical properties, hydrogels hold considerable promise for biomedicine and soft devices. Yet, many applications demand that they not only sustain mechanical loads but also maintain low friction and wear resistance at dynamic interfaces. Consequently, integrating high toughness with low friction is essential for deploying hydrogels under coupled loading–sliding conditions. This chapter surveys recent advances in five key application areas: tissue engineering, device coatings, drug delivery, electronic devices, and soft actuators.

### 4.1. Tissue Engineering

Natural soft tissues—such as articular cartilage, cornea, tendons, and heart valves—routinely experience dynamic mechanical loads under physiological conditions. Their microstructures and surface properties have evolved over time to synergistically combine high toughness with low friction, providing a well-defined biomimetic blueprint for tough, low-friction hydrogels in tissue engineering.

#### 4.1.1. Cartilage Tissue Engineering

**Figure 9:**
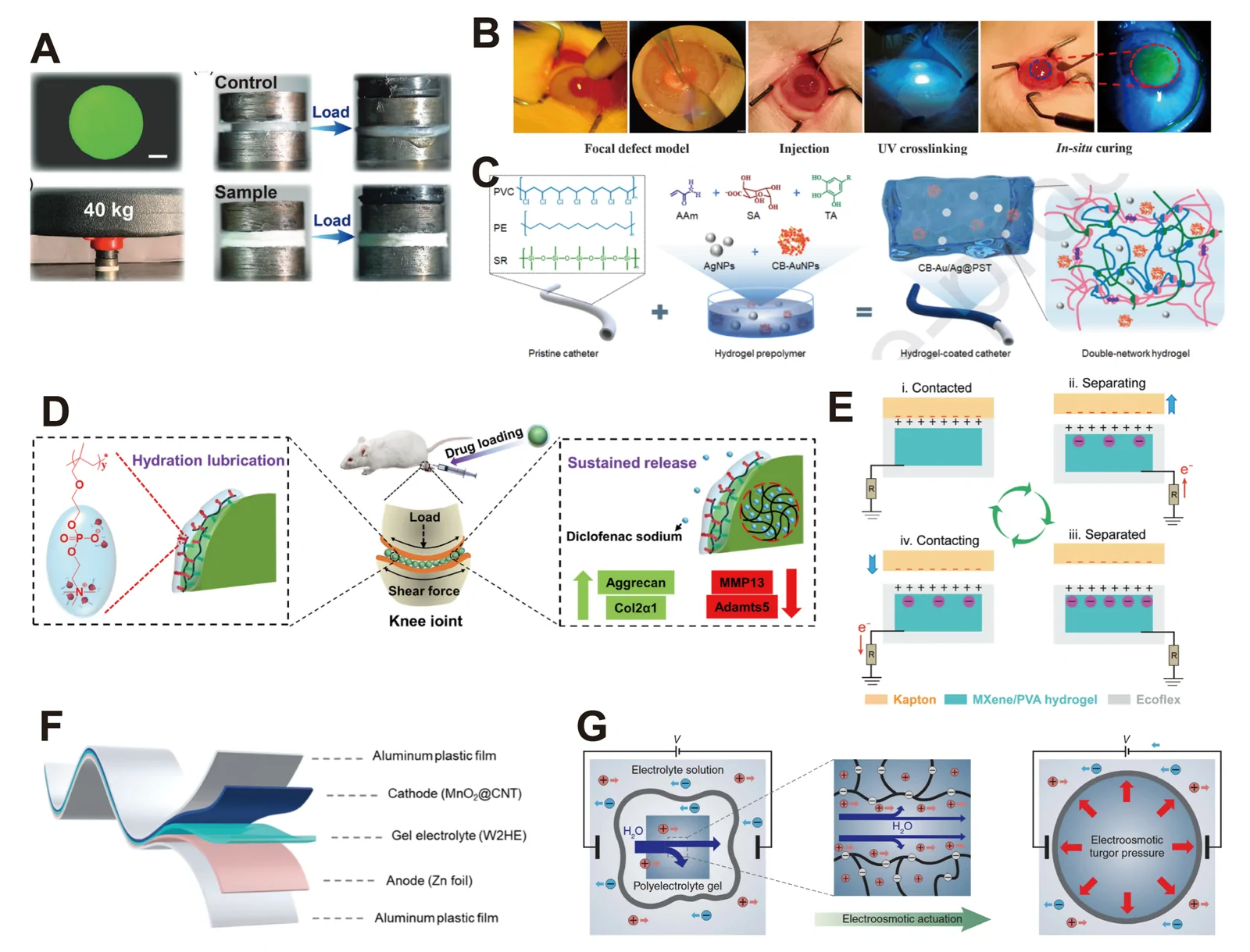
Applications of Tough, Low-Friction Hydrogels. (**A**) Cartilage tissue. Reproduced with permission [104]. Copyright 2024, Wiley. (**B**) Corneal tissue. Reproduced with permission [105]. Copyright 2023, Elsevier. (**C**) Device coatings. Reproduced with permission [106]. Copyright 2024, Elsevier. (**D**). Drug delivery Reproduced with permission [107]. Copyright 2025, Elsevier. (**E**) Energy harvesting. Reproduced with permission [108]. Copyright 2021, Wiley. (**F**) Hydrogel batteries. Reproduced with permission [109]. Copyright 2023 ACS. (**G**) Soft actuators. Reproduced with permission [110]. Copyright 2022, AAAS.

Articular cartilage is a sparsely cellular, avascular tissue with a stratified architecture whose primary functions are to provide a low-friction sliding surface and to transmit loads to the subchondral bone [89]. These functions are enabled by a highly coordinated design: negatively charged aggrecan osmotically attracts water, generating swelling pressure, which is confined by the dense type II collagen fibril network [97]. This swelling–confinement balance imparts cartilage with its unique combination of load-bearing capacity and lubrication. In healthy joints, surface phosphatidylcholine lipids, together with hyaluronic acid, lubricin, and aggrecan, assemble into a complex hydration lubrication layer [98]. Through charge–dipole interactions, this layer anchors abundant water molecules at the sliding interface, maintaining the coefficient of friction (COF) at an ultra-low 0.001–0.03 even under physiological contact stresses of 1–20 MPa [99,100]. Because cartilage is avascular, aneural, and lacks lymphatic drainage, its intrinsic repair capacity is extremely limited [101]; developing repair materials that integrate mechanical support with surface lubrication therefore represents a critical challenge. Endowing a single hydrogel system with long-term fatigue resistance, low wear, and appropriate biocompatibility on par with native cartilage remains formidable. While traditional tough hydrogels have advanced substantially in mechanical strength, their lubrication performance remains inadequate. PAMPS/PDMAAm double-network (DN) hydrogels, a representative tough system, have shown potential for cartilage repair: after one million friction cycles, the maximum wear depth of this DN gel was only 3.20 μm, matching that of clinically used UHMWPE (3.33 μm) [48], and when implanted into rabbit osteochondral defects, it induced hyaline cartilage-like repair tissue rich in type II collagen and aggrecan [102]. Zhao et al. [103] introduced a strategy based on electrostatically crosslinked crystalline domains. Using sodium tripolyphosphate to simultaneously achieve electrostatic crosslinking and in situ salting-out, they prepared a super-anti-swelling hydrogel whose swelling ratio remained below 2% after 460 days of phosphate-buffered saline (PBS) immersion. Under a contact pressure of 2.8 MPa, this gel exhibited a COF of 0.0084 and endured 100 000 sliding cycles; it could also serve as a robust lubricating coating on artificial joint components such as ceramics and titanium alloys, reaching an interfacial toughness up to 920 J/m^2^. Zhao et al. [104] further created a cartilage-mimetic hydrogel by covalently grafting a thick polyelectrolyte brush layer onto the subsurface of a tough matrix with a multiscale crystalline architecture. Under high contact pressure, this hydrogel sustained durable lubricity over 100,000 reciprocating sliding cycles, outperforming natural animal cartilage (Figure 9A). This series of advances demonstrates that combining surface brush layers with highly fatigue-resistant bulk networks can approach, and in some metrics surpass, the tribological performance of native cartilage.

#### 4.1.2. Corneal Tissue Engineering

The cornea is a layered, avascular connective tissue that is highly transparent. As a key component of the ocular refractive system, it must simultaneously provide optical clarity, mechanical integrity, and lubrication at the eyelid–cornea interface [111]. Hierarchically organized collagen fibrils in the stroma confer both toughness and transparency, while the tear film and glycocalyx on the corneal epithelium supply surface lubrication. Globally, approximately 12 million patients suffer from corneal blindness—yet donor cornea shortages leave them untreated—making the development of mechanically compatible, optically transparent, and surface-lubricating corneal substitutes an urgent priority [112]. Hydrogels for corneal tissue engineering must therefore satisfy three essential requirements: transparency, toughness to withstand suturing and intraocular pressure, and sufficient surface lubrication to permit smooth eyelid gliding and support healthy epithelial cell spreading. Researchers have made notable strides toward these goals. Wang et al. [105] developed an injectable double-network (IDN) hydrogel from methacrylated gelatin (Gel-MA) and oxidized hyaluronic acid (OHA) that cures in situ via Schiff base reaction and UV crosslinking, enabling the repair of focal corneal defects. The elastic modulus, tunable by adjusting the Gel-MA-to-OHA ratio from 3.4 to 176.4 kPa, matches that of native cornea. In a rabbit epithelial–stromal defect model, the IDN hydrogel group achieved complete corneal epithelial regeneration and orderly stromal reconstruction by day 35 (Figure 9B), without significant scar formation. To attain mechanical compatibility with native cornea while guiding orderly stromal regeneration, Kong et al. [113] used near-field direct-writing electrospinning to fabricate orthogonally aligned poly(ε-caprolactone)-polyethylene glycol (PECL) submicron fiber meshes, which were then infused with GelMA hydrogel to create a fiber-reinforced composite. By fine-tuning the fiber spacing, the 100G configuration (100 μm spacing) yielded overall properties closest to native cornea: tensile strength 3.47 ± 0.43 MPa, compressive modulus 121.3 ± 10.2 kPa, and light transmittance 74.7% at 500 nm. The aligned fiber topography induced limbal stromal stem cells to maintain a keratocyte phenotype, suppressing fibroblast transformation even in serum-containing media and guiding the secretion of organized type VI collagen characteristic of keratocytes. Li et al. [114] introduced a “T.E.S.T.” corneal patch designed to satisfy four criteria: Transparency, Epithelium & Stroma generation, Suturelessness, and Toughness. They incorporated bifunctional micelles, co-self-assembled from a photo-crosslinkable toughening component (F127DA) and an aldehyde-functionalized adhesive component (AF127), into GelMA hydrogel, and combined them with corneal crosslinking (CXL). These micelles markedly enhanced the hydrogel toughness (elongation at break nearly 600%), while the addition of type I collagen formed an auxiliary CXL network, raising the burst pressure above 400 mmHg—far exceeding normal intraocular pressure (10–21 mmHg). The patch can be photocured in situ, filling corneal defects, tightly adhering to the stromal bed, and promoting epithelial and stromal regeneration. These three strategies illustrate a clear trajectory: from injectable in situ curing to fiber-reinforced composite to adhesive patch, each progressively integrates toughness, transparency, and interfacial lubrication, collectively advancing corneal hydrogel substitutes toward clinical translation.

### 4.2. Device Coatings

Implantable and interventional medical devices—such as catheters, vascular stents, prosthetic heart valves, and neural electrodes—maintain persistent contact with biological tissues or body fluids during service. The stability of these interfaces directly governs device lifespan: the coating must resist deformation and wear induced by tissue motion or device manipulation, preventing crack propagation and delamination [115], while its surface must exhibit low friction to minimize insertion trauma and inhibit protein and microbial adhesion, thereby reducing infection risk [116]. Conventional hydrophilic coatings can provide short-term lubrication and fouling resistance through a physically adsorbed hydration layer, but their weak substrate adhesion and rapid wear under repeated sliding severely compromise long-term performance. Grafted polymer coatings are susceptible to shear-induced damage and delamination, and have yet to achieve tissue-mimetic water content and mechanical compliance [117]. These limitations have driven interest in hydrogel coating systems that combine high interfacial toughness with intrinsic fatigue resistance. Liu et al. [106] developed a CB-Au/Ag@PST hydrogel coating with water-responsive Janus adhesion and acidity-triggered transition (Figure 9C), composed of a PAAm/ sodium alginate (SA)/tannic acid (TA) double-network matrix, silver nanoparticles (AgNPs), and chitosan/ bovine serum albumin (BSA)–gold nanoparticles chitosan/bovine serum albumin–gold nanoparticles (CB-AuNPs). This coating exhibited an adhesion strength of 44.6 ± 1.9 kPa on PVC, PE, and silicone rubber catheter substrates, preventing detachment during insertion. Upon water exposure, surface hydrophilic groups rapidly form a hydration layer, reducing the coefficient of friction from ~0.60 to ~0.03 and substantially alleviating friction-induced pain during catheterization.

Zwitterionic polymers have attracted considerable interest for medical device coatings owing to their exceptional antifouling and lubricating properties. Wang et al. [118] designed a polyelectrolyte–polyzwitterion bilayer hydrogel coating (PAE-PSV) consisting of a PEI/PAA adhesive underlayer and a PSBMA/PolySBMA zwitterionic top layer, chemically bonded via N-succinimidyl acrylate to reinforce interfacial adhesion. Reversible electrostatic interactions within the underlayer endow the coating with outstanding flexibility (elongation at break > 1000%) and self-healing capacity. The zwitterionic top layer forms a dense hydration layer through ionic solvation, reducing the water contact angle to 9–13° and imparting excellent lubricity. This hydration layer also resists nonspecific protein adsorption, suppresses L929 cell and platelet adhesion, and prolongs clotting time.

Beyond biomedical devices, hydrogel coatings have also been developed for engineering applications that demand extreme environmental tolerance and sustained drag reduction under harsh conditions. Unlike medical coatings, which prioritize biocompatibility and physiological interface stability, engineering coatings must resist wide temperature fluctuations, freeze–thaw cycles, and prolonged mechanical abrasion. Li et al. [119] developed a lubricating organohydrogel comprising a poly(ethylene glycol)–polydimethylsiloxane (PEG–PDMS) network infused with methyl silicone oil (MSO). The immiscibility of MSO with the polymer matrix induces interfacial phase separation, driving the PEG–PDMS chains into an aligned, tightly packed arrangement and elevating the tensile strength to 1.38 MPa. Separately, grooved surface textures mechanically lock the lubricant under shear, enabling self-replenishing lubrication. The coating achieved a steady-state coefficient of friction (COF) of 0.08 and sustained over 18,000 friction cycles, with an operational window from −40 °C to 150 °C and negligible friction variation across this range. As a drag-reduction coating, it markedly lowered ice adhesion and allowed icebreakers to traverse ice masses with substantially reduced resistance. This work establishes molecular alignment—driven by lubricant–matrix immiscibility—as a design principle for reconciling lubricant-induced softening with long-term wear resistance under extreme environments, positioning organohydrogel coatings as promising candidates for energy-saving and anti-icing applications in polar, deep-sea, and aerospace engineering.

### 4.3. Drug Delivery

By virtue of their high water content and tunable permeability, hydrogels can encapsulate and control the release of diverse hydrophilic drugs, proteins, and even living cells [120]. Imparting low-friction characteristics to mechanically robust drug-delivery hydrogels helps protect surrounding tissues from wear-induced damage at sliding interfaces, while the formation of a hydration lubrication layer inhibits nonspecific protein adsorption, collectively prolonging the service life of the delivery system. Zhang et al. [121] photopolymerized methacrylated gelatin (GelMA), acrylamide (AM), and 2-methacryloyloxyethyl phosphorylcholine (MPC) to form a GelMA-PAM-PMPC composite hydrogel. The phosphorylcholine groups anchor abundant water molecules at the sliding interface via charge–dipole interactions, creating a hydration shell that resists squeeze-out under normal load yet relaxes readily under shear to yield a fluid-like response; this reduced the coefficient of friction (COF) from 0.052 (without MPC) to 0.011 at 30% MPC content. The COF remained stable over 10 000 reciprocating cycles, demonstrating durable lubricity. Concurrently, the hydrogel achieved an encapsulation efficiency of 49.2% for diclofenac sodium and a cumulative release of 64.5% over 10 days, realizing sustained drug delivery. Han et al. [122] fabricated GelMA microspheres via microfluidics and coated them with a dopamine–phosphorylcholine copolymer (DMA-MPC), endowing the microspheres with both enhanced lubrication and controlled drug release. Guo et al. [107] developed an injectable hyaluronic acid composite hydrogel (HPD/Met@GMs) based on phenylboronic acid–catechol dynamic crosslinking. Leveraging its shear-thinning behavior for intra-articular injection, the hydrogel provides ROS scavenging, MMP-9-responsive sustained release of metformin, and a reduced COF of 0.049–0.080. In a rat osteoarthritis model, it afforded significant protection against cartilage matrix degradation (Figure 9D). These examples illustrate how integrating hydration-lubricating moieties with tough, drug-eluting networks can yield multifunctional delivery platforms that simultaneously protect sliding interfaces and sustain therapeutic release.

Endowing drug-delivery hydrogels with environmental responsiveness enables on-demand release and precise dose control. Liang et al. [12] systematically investigated the role of cellulose nanofibril (CNF)- and chitosan-reinforced polyethylene glycol/sodium acrylate composite hydrogels in controlled drug release. CNF forms a rigid, dense network through hydrogen-bonding physical crosslinks and covalent carbamate bonds, restricting chain mobility; under alkaline conditions (pH 9), the release of Rhodamine B exceeded 89.6% within 21 days, demonstrating pronounced pH-dependent release behavior. By contrast, chitosan creates a chemically reinforced network via amidation of its amino groups with the matrix and Ca^2+^ coordination crosslinks; the protonation–deprotonation equilibrium imparts a more gradual release profile, with release reaching approximately 75% across all pH conditions tested. In high-ionic-strength environments, release from both gels accelerated markedly, with that from the chitosan gel increasing from 64.2% to 90.2%. Oliveira et al. [123] loaded diclofenac sodium or ketorolac into PVA hydrogels annealed at 150 °C and controlled release using vitamin E and the cationic surfactant cetalkonium chloride. Hydrophobic vitamin E aggregates created a drug-diffusion barrier, whereas cetalkonium chloride bound electrostatically to the anionic drugs, thereby transforming the release from an initial burst to sustained delivery within 24 h and producing a particularly gradual profile for ketorolac.

### 4.4. Electronic Energy-Harvesting and Storage Devices

The rapid evolution of flexible electronics—from electronic skin and wearable health monitors to soft robotics—has imposed revolutionary demands on the flexibility, conductivity, and mechanical reliability of power supply units [124]. With their tunable ionic conductivity, intrinsic compliance, and mechanical compatibility with biological tissues, hydrogels have emerged as ideal electrolyte and electrode materials for flexible batteries, supercapacitors, and triboelectric nanogenerators (TENGs). The operation of hydrogel-based TENGs relies on the synergy of contact electrification and electrostatic induction. [125] The low-friction, low-adhesion hydrogel surface can suppress parasitic energy dissipation from stick–slip detachment and facilitate cleaner contact–separation cycles; however, the degree to which this enhances the effective separation area and charge-transfer efficiency is device-dependent and must be evaluated together with the effective contact area, material pairing, interfacial charge density, and operating conditions. Simultaneously, the high strength, toughness, and fatigue resistance of the hydrogel enable it to resist stress concentration and impede crack propagation under prolonged cyclic loading, preserving the structural integrity of both the ionic conduction network and the triboelectric interface. Luo et al. [108] developed a flexible multifunctional TENG based on an MXene/PVA hydrogel (MH-TENG). The MXene nanosheets facilitate PVA crosslinking and create microchannels within the hydrogel, enhancing ion transport during the triboelectric process (Figure 9E). At 4% MXene doping, the single-electrode-mode device delivered an open-circuit voltage of 230 V, a short-circuit current of 270 nA, and a transferred charge of 38 nC, representing an approximately fourfold enhancement over undoped PVA. Chen et al. [126] designed an ionized cellulose (IMC) by covalently grafting an L-histidine-based ionic liquid onto cellulose chains and then copolymerizing it with acrylamide (AM) to construct an ion-conducting IMC/PAM double-network hydrogel featuring both physical and chemical crosslinks. Strong ion–dipole interactions between the imidazolium cations, carboxylate anions, and the amide groups of AM endow the hydrogel with robust mechanical properties (tensile strain 752 ± 12.1%, tensile strength 0.23 ± 0.03 MPa) and appreciable ionic conductivity (0.045 S/m). As a single-electrode TENG electrode, this hydrogel outputs 25 V under a 20 N load at a power density of 24.9 mW/m^2^, with no performance degradation after 1100 cycles. Beyond mechanical-to-electrical conversion via triboelectric nanogenerators, moisture-driven energy generation (MEG) offers an alternative route for harvesting ambient energy, particularly in wearable and self-powered systems. However, conventional hydrogel-based MEGs suffer from rapid water loss and performance degradation under low-humidity or fluctuating conditions. Zhao et al. [127] addressed this by stacking a negatively charged MXene (Ti_3_C_2_T_x_) aerogel onto a polyacrylamide hydrogel and converting the latter to an organohydrogel via ethylene glycol (EG) solvent exchange. This EG substitution dramatically improved water retention: the organohydrogel lost less than 1 wt% over 6 days at 20% RH, whereas the pristine hydrogel shrank to 70% within 2 days. By preserving the internal ionic concentration gradient, this robust hydration stability enabled a stable open-circuit voltage of ~430 mV for over 15 days across a wide relative humidity range (20–95%) and a broad temperature window (−20 to 80 °C). The device delivered a maximum power density of ~10 μW cm^−2^ and, when connected in series, powered commercial calculators and LEDs even on rainy or hot days. Notably, the porous structure of the MXene aerogel facilitated selective Ca^2+^ diffusion, as confirmed by Density Functional Theory (DFT) calculations and Kelvin probe force microscopy. This work demonstrates that solvent displacement not only prevents dehydration-induced failure but also sustains the electrokinetic charge-separation mechanism, offering a promising strategy for long-term, environment-tolerant energy harvesters that complement conventional TENG and battery systems in wearable electronics.

For energy storage, conventional hydrogel electrolytes undergo sustained cyclic loading during charge–discharge cycling, which nucleates and propagates cracks, ultimately causing failure of both the hydrogel and the battery [128]. To circumvent this, researchers have developed a nanoporous hydrogel thermocell that exploits the synergy between co-nonsolvency and the Hofmeister effect [129]. The interconnected nanoporous network furnishes highly efficient ion-transport channels, delivering a high thermoelectric power of 4.13 mV K^−1^, an ultrahigh ionic conductivity of 11.07 S m^−1^, and a normalized output power density of 5.34 mW m^−2^ K^−2^. Concurrently, the densified porous network backbone imparts mechanical robustness, with a tensile strength of 9.06 MPa and an elongation of 1460%. Connecting 20 thermoelectric units in series generates a voltage exceeding 2 V—sufficient to directly power electronic devices—underscoring the considerable potential for thermoelectric conversion and self-powered flexible technologies. He et al. [109] prepared a densely entangled polyacrylamide hydrogel electrolyte by using a high monomer concentration and an extremely low crosslinker content (Figure 9F), thereby achieving a combination of high tensile strength (446 kPa), high elastic modulus (128 kPa), and high ionic conductivity (3.93 mS cm^−1^), and breaking the trade-off between mechanical properties and ionic conductivity. This highly entangled hydrogel leverages interfacial confinement to regulate the nucleation and growth of zinc hydroxide sulfate (ZHS) on the zinc anode surface in situ, forming fine, uniform, substrate-parallel lamellar structures that homogenize the electric field and suppress zinc dendrite growth. A zinc anode employing this electrolyte exhibits long-term cycling stability over 6000 h at 0.5 mA cm^−2^ and operates stably for more than 220 h even at a high depth of discharge of 68.4%. The assembled quasi-solid-state Zn//MnO_2_ pouch cells and fiber cells exhibit excellent flexibility and cycling stability (99.76% capacity retention after 100 cycles) and reliably supply power under harsh conditions such as bending, cutting, and water immersion, offering a new electrolyte design paradigm for wearable energy storage devices.

Beyond energy harvesting and storage, integrating high mechanical toughness, environmental stability, and ionic conductivity in hydrogels is essential for next-generation flexible wearable sensors. In this context, organo-hydrogels have emerged as a promising platform. Through a solvent-displacement strategy, they inherit the excellent mechanical properties of conventional hydrogels while overcoming their notoriously poor environmental adaptability. Gao et al. [130] combined a dynamic hydrophobic association network with a glycerol solvent-displacement strategy to prepare a conductive organo-hydrogel that simultaneously delivers ultrahigh stretchability (3501%), high toughness (12.87 MJ m^−3^), and room-temperature self-healing. The glycerol/water binary solvent imparts stable ionic conductivity over a broad temperature range (−20 to 60 °C)—reaching 0.06 S m^−1^ even at −20 °C—and exceptional water retention (92% mass retention after 7 days in an open environment). A single-electrode triboelectric nanogenerator (TENG) fabricated from this organo-hydrogel exhibits an open-circuit voltage of 127 V, a power density of 205 mW m^−2^, and negligible output degradation after 10 000 cycles. More importantly, integrating such self-powered TENG sensors with machine learning (e.g., long short-term memory, LSTM) enables accurate material identification with a recognition accuracy of 99.3%. This work demonstrates that designing organo-hydrogels via a solvent-displacement strategy can unify high mechanical toughness, wide-temperature-range stability, and self-powered sensing, thereby paving the way for sustainable, environmentally adaptive wearable electronics and intelligent sensing systems.

### 4.5. Soft Actuators

Hydrogel actuators are smart soft materials that undergo large volumetric or shape changes in response to external stimuli such as temperature, pH, light, electric fields, and humidity. Compared with conventional rigid actuators, they offer unique advantages—tissue-like compliance, high water content, and biocompatibility—that enable high-curvature bending and twisting in confined spaces [131]. To function reliably, these actuators must combine high strength, toughness, and fatigue resistance to deliver reproducible force output and resist tearing under repeated large deformations, while simultaneously presenting ultra-low and dynamically tunable friction at their surfaces. Moreover, most existing hydrogel actuators rely on osmosis-driven water diffusion, resulting in inherently slow response kinetics. Developing tough and intelligent hydrogel actuators that integrate rapid response, large actuation force, and long-term operational stability therefore remains a critical challenge in the field.

The slow response of hydrogel actuators stems fundamentally from passive water diffusion and the sluggish conformational relaxation of polymer networks. Recent advances have achieved simultaneous gains in speed and force output by directionally controlling water transport and network energy-dissipation pathways. At the water-transport level, Na et al. [110] replaced passive osmosis with electric-field-driven electro-osmotic flow, in which migrating ions carry water molecules, shifting mass transport from diffusion to convection (Figure 9G); this generated an actuation stress of 0.79 MPa within 9 min and fundamentally overcame the diffusion-limited rate bottleneck. Zeng et al. [132] exploited anisotropic swelling induced by a large-span crosslinking gradient to funnel the random motion of water molecules into a directional expansive force, achieving a bending rate as high as 190° s^−1^. At the network-relaxation level, Zha et al. [133] advanced this concept by exploiting a solvent-exchange-triggered hydrogen-bond activation strategy in a poly(vinyl alcohol) (PVA)/tannic acid (TA) nanofibrillar organohydrogel, achieving a response rate of 0.25% s^−1^ with excellent cyclic stability while simultaneously resolving the mechanical deterioration typical of hydrogel actuation. In the precursor state, glycerol preferentially hydrogen-bonds with TA, suppressing TA–PVA crosslinking and maintaining a loose network. Upon immersion in deionized water, selective glycerol release coupled with water retention triggers hydrogen-bond activation between PVA and TA, inducing dense physical crosslinks that drive an anomalous volume contraction to 56.3% of the original volume—opposite to the swelling behavior of conventional hydrogels. This transformation converts the initially loose network into a highly entangled, porous nanofibrillar architecture, elevating the tensile strength to 5.4 ± 0.2 MPa, fracture energy to 134.9 ± 7.6 kJ/m^2^, and toughness to 18.5 ± 0.7 MJ/m^3^—substantially outperforming typical hydrogel actuators that degrade mechanically upon hydration. Leveraging this mechanism, twisted multi-filament SOA fibers exhibited rapid contraction upon water immersion (0.25% s^−1^) with a specific power of 0.28 W/kg^1^, and further displayed humidity-sensitive reversible actuation at 80% relative humidity, with an angle change rate of 3.1° min^−1^ and excellent repeatability over multiple cycles. Critically, this solvent-exchange strategy establishes a conceptually new design paradigm: solvent composition serves as a transient regulator of crosslinking density rather than merely a passive swelling medium, thereby resolving the long-standing conflict between actuation stroke and mechanical integrity in hydrogel-based artificial muscles. This work also highlights the broader potential of organohydrogels in soft robotics, where environmental adaptiveness (anti-freezing and anti-dehydration) can be intrinsically combined with high-force output—a combination rarely achieved in conventional water-based hydrogel actuators. Yao et al. [134] devised an elastic-potential-energy decoupling strategy: energy is pre-stored by locking the network in a pre-stretched state, and subsequent thermal dissociation of crosslinks at 80 °C triggers the instantaneous release of that stored energy, compressing network relaxation into a single burst and producing a contraction stress of 850 kPa within 90 s, effectively reconciling the inherent trade-off between speed and force output.

The functionality of hydrogel actuators extends beyond deformation output; interfacial interactions between the actuator and the target object directly govern manipulation capability. A representative example is the work of Deng et al. [135]: inspired by the dynamic transition of oral mucosa from lubrication to astringency, they introduced mucin into a poly(vinyl alcohol) network. The charged oligosaccharide side chains of mucin form a dense hydration lubrication layer, reducing the surface friction coefficient to approximately 0.009. Upon exposure to tannic acid, hydrophobic and hydrogen-bonding interactions between mucin and polyphenols induce precipitation and dehydration, causing the friction coefficient to rise sharply to approximately 0.47—a 52-fold switching amplitude with good reversibility. By integrating surface lubrication as an independently tunable dimension into hydrogel actuator design, this work enables the same material system to reversibly switch between “low-friction release” and “high-friction gripping” states, offering a straightforward and versatile interfacial engineering strategy for adaptive soft grippers.

## 5. Conclusions

Despite the immense potential hydrogels have demonstrated across diverse fields, they are confronted by a persistent and fundamental contradiction: the bulk energy-dissipation mechanisms essential for toughening intrinsically interfere with—and often disrupt—the stable surface hydration layer required for lubrication, making it exceptionally difficult to reconcile strength and toughness with low friction and wear resistance within a single material system. This incompatibility severely compromises long-term service reliability under dynamic loading, rendering the translation of laboratory performance metrics into robust, real-world device functionality a formidable challenge.

In recent years, a suite of innovative toughening and friction-reduction strategies has dramatically expanded the performance boundaries of hydrogels. Double networks, dynamic bonds, and nanocomposites have elevated hydrogel fracture strength from hundreds of kilopascals to tens of megapascals; surface polymer brushes, lipid-based lubrication layers, and structured hydration films have driven the friction coefficient down to as low as 0.001—rivaling or even surpassing the boundary lubrication coefficient of natural articular cartilage (0.002–0.02). With the progressive integration of functionalities such as self-healing and tear resistance, these materials are increasingly matching or exceeding natural biological benchmarks in overall mechanical performance.

This review has sought to articulate the design principles and synergistic mechanisms of tough, low-friction hydrogels. The central tenet is functional decoupling of bulk dissipation from surface lubrication: the bulk network dissipates external work through multi-scale structural design and dynamic-bond reorganization, while a highly hydrated surface polymer brush or boundary lubrication layer concurrently lowers shear resistance, ensuring that energy dissipation and interfacial shear operate with spatial and mechanistic independence. This decoupling breaks the conventional toughness–lubrication trade-off. Yet, translating these advances into materials that reliably meet complex application demands requires that several persistent challenges be critically addressed. First, although double-network and sacrificial-bond dissipation strategies can dramatically enhance toughness, the pronounced hysteresis they generate under cyclic shear produces undesirable heating that is expected to threaten surface hydration-layer stability and thereby contribute to friction escalation. While this thermo-mechanical coupling is physically reasonable, its quantitative dominance relative to other friction-rise mechanisms—such as debris accumulation or contact-area evolution—in specific hydrogel systems and operating windows remains to be established. Second, while dynamic bonds and self-healing mechanisms offer the potential to repair worn surfaces, the kinetic competition between bond re-formation and material removal during sliding—documented in Schiff-base and boronate-ester systems under reciprocating contact [52,54]—highlights a fundamental constraint: the characteristic timescales of dynamic bond exchange (milliseconds to seconds) can be insufficient relative to the rate of surface material removal under high-shear tribological conditions. Whether this mismatch can be overcome by accelerating bond kinetics (e.g., via catalysis, local heating, or mechanochemical activation) or whether it represents an intrinsic limitation of current dynamic chemistries remains a critical open question. In either case, making rapid recovery of surface hydration and rehydration an urgent technological priority. Third, the long-term durability of these hydrogels under physiologically relevant cyclic loading remains insufficiently validated. Most reported wear tests are limited to 10^4^–10^6^ cycles, far short of the 10^6^–10^8^ cycles typical of natural joint motion over a human lifetime. Systematic studies on fatigue crack propagation under combined mechanical and tribological loading—where wear and fatigue synergistically accelerate material failure—are urgently needed. Fourth, the environmental stability of tough, low-friction hydrogels remains inadequate: swelling under humid conditions dilutes the network and strips away lubricating moieties, whereas dehydration eliminates hydration lubrication altogether, collectively compromising long-term mechanical and tribological reliability. Critically, classical Stribeck curves and boundary lubrication models have yet to incorporate the damage mechanisms—network fatigue fracture, wear debris generation, and transfer—that dominate under cyclic friction. Consequently, quantitative prediction of friction life from intrinsic network structure and operating conditions remains a largely uncharted territory.

Beyond these scientific challenges, practical translational barriers are equally formidable. First, the scalability and reproducibility of current fabrication strategies—particularly surface-initiated polymerization, gradient structures, and porous scaffold infiltration—remain largely unaddressed. Most syntheses are limited to laboratory-scale batch processes with low throughput, and batch-to-batch consistency in mechanical and tribological properties has rarely been reported. Second, economic viability is a pressing concern: costly components (specialized monomers, noble metal nanoparticles, complex crosslinkers) and multi-step processing routes substantially escalate production expenses. Third, biomedical applications face stringent regulatory requirements, including ISO 10993 biocompatibility testing [136], sterilization compatibility with hydrogel integrity, and comprehensive characterization of long-term in vivo degradation and immune responses. To date, few tough, low-friction hydrogels have advanced to systematic preclinical evaluation, and none have obtained regulatory clearance for clinical use.

Meanwhile, applications in tissue engineering, drug delivery, electronic energy-harvesting and storage devices are demanding more than a single function: beyond low friction and high load-bearing capacity, these scenarios often require materials that simultaneously integrate electrical conductivity, bioactivity, controlled release, and degradability. Future research will likely focus on novel multifunctional synergy mechanisms, in-depth structure–property relationships, and fabrication strategies that combine stability under extreme environments with intelligent responsiveness. As research continues to deepen, these increasingly sophisticated tough, low-friction hydrogels are poised to exert profound impact across a far broader range of applications.

## Figures and Tables

**Table 1:** Comparison of representative design strategies for strong, tough and low-friction hydrogels.

Representative System	Advantages	Limitations	Core Design Principle
Double-network (DN)	High fracture energy, high compressive strength	Large hysteresis, debris aggravates wear	Brittle network fractures to dissipate energy; ductile network sustains deformation
Self-healing/dynamic bond	Interfacial self-repair, extended service life	Repair kinetics may lag wear; environmentally sensitive	Reversible bonds rearrange under shear to repair interfacial damage
Nanocomposite/fiber-reinforced	High strength, high modulus, anisotropy	Exposed particles aggravate abrasive wear	Fillers/fibers transfer load, deflect cracks, and bridge
Topological/highly entangled	Low hysteresis, high fatigue threshold	Demanding synthesis control	Entanglement points slide to homogenize stress, avoiding irreversible rupture
Vertical gradient	One-step fabrication, natural partitioning	Precise gradient control challenging	Loose surface for lubrication; dense bulk for load-bearing
Bilayer heterogeneous (bulk + brushes)	Independent optimization of surface and bulk	Complex grafting, interfacial adhesion critical	Surface hydrated brushes lubricate; tough bulk bears load
Porous scaffold infiltration	Reinforcement isolated from friction interface	Requires continuous replenishment of mobile chains	Scaffold bears load; mobile chains provide boundary lubrication

## Data Availability

This review article does not involve the creation or analysis of new experimental data. All data discussed are derived from previously published studies, which are cited and referenced throughout the manuscript. Data sharing is not applicable to this article.

## Supplementary Materials

The following supporting information can be downloaded at: https://www.mdpi.com/article/10.3390/gels12090775/s1.
