# Supplementary material for: Design and Application of Strong and Tough Low-Friction Hydrogels
Source: Gels. 2026 Aug 30;12(9):775. doi: 10.3390/gels12090775 (PMC13606718; doi:10.3390/gels12090775)
Supplement: Supplementary file 1 [file gels-12-00775-s001.zip › gels-4494268-supplementary.pdf]

**Supplementary Table S1**. Detailed tribological and mechanical parameters of representative tough hydrogels.

| Ref. | Hydrogel System | Counterface | Load/Contact Pressure | Speed | Lubricant | Duration/Cycles | COF | Corresponding Wear | Bulk Mechanical Properties |
|---|---|---|---|---|---|---|---|---|---|
| [48] | PAMPS-PDMAAm DN | Alumina pin | 4.7 N | 50 mm/s | Distilled water | $10^6$ cycles | ~0.12 | Max wear depth 3.20 μm | Modulus 0.20 MPa; Strength 3.1 MPa |
| [49] | DN gel film (1.5 mm) | SiC disc | Not specified | Sliding in water | Water | >2 run-in cycles | <0.01 | Not reported | Contact stiffness 9.83 N/mm |
| [50] | DN with zwitterionic 2nd network (MEDSAH) | Steel ball | 10 mN | 20 mm/s | Deionized water | Speed-dependent | ~0.01 | Not reported | Surface modulus ~423 kPa |
| [52] | Self-healing Schiff-base DN coating on Ti6Al4V | $Si_3N_4$ ball | 2 N | 1 Hz | SBF | 1800 s | 0.130 | Significantly lower than bare Ti6Al4V | Adhesion 22.5 N; G'~$10^4$ Pa; self-healing |
| [56] | Silica NP–alginate–PAAm NC (4 wt% $SiO_2$) | Alumina ball | 0.7 N | 100 mm/s | Bovine serum | 1000 m | 0.0035 | Wear volume reduced >69% | Compressive modulus 0.65–1 MPa |
| [58] | Concrete-like PVA/PEG (BPP hydrogel) | Stainless steel ball | 1 N | 5 Hz | HA–plasma mixture | 18,000 s | ~0.005 | Groove: 416.7×68.8 μm | Compressive 29.5 MPa; Tensile 10.5 MPa; Fatigue retention 91.4% |
| [69] | PIE DN (highly entangled) | N/A (bulk study) | N/A | N/A | N/A | N/A | N/A | N/A | Strength 1.25 MPa; Toughness 5800 J/m²; Hysteresis <0.15 |
| [96] | DN + grafted PMETAC brushes | Glass hemisphere | 1 N | 0.1 mm/s | Deionized water | ⩾6 cycles | 0.001 | Negligible | Surface E ~1.7 MPa; Water content >95% |
| [94] | Gradient PAA-Fe/PAM + comb-like lubricant | Steel cylinder | 20 N | 1 Hz | Deionized water | 36,000 cycles | 0.0028 | Slight wear (surface layer) | Compressive modulus ~15 MPa; Water content ~60.7% |
| [90] | Macroporous PAAm + alginate chains | Glass plate | ~$10^5$ Pa | 0.1 rad/s | Alginate solution | 6 h | ~0.008 | Slight scratches only | Pore size ~50 μm; Water content ~96% |

Notes: "Optimal" refers to the single condition yielding the lowest steady-state COF or best wear resistance in each study. Direct cross-comparison across studies is not advised due to different test setups. Ref. numbers correspond to main text. N/A = not applicable/not tested. For [58], COF estimated from Fig. 4o.
